# Water and Carbon Dioxide Capillary Bridges in Nanoscale Slit Pores: Effects of Temperature, Pressure, and Salt Concentration on the Water Contact Angle

**DOI:** 10.1021/acs.langmuir.4c01185

**Published:** 2024-08-19

**Authors:** Arthur Prado Camargo, Arben Jusufi, Alex Gk Lee, Joel Koplik, Jeffrey F. Morris, Nicolas Giovambattista

**Affiliations:** † Instituto de Física, Universidade de São Paulo, 05508-090 Sao Paulo, SP, Brasil; ‡ ExxonMobil Technology and Engineering Company, 1545 US Rt. 22 East, Annandale, New Jersey 08801, United States; § Levich Institute, City College of New York, New York, New York 10031, United States; ∥ Department of Physics, City College of New York, New York, New York 10031, United States; ⊥ Ph.D. Program in Physics, The Graduate Center of the City University of New York, New York, New York 10016, United States; # Department of Chemical Engineering, City College of New York, New York, New York 10031, United States; ∇ Department of Physics, Brooklyn College of the City University of New York, Brooklyn, New York 11210, United States

## Abstract

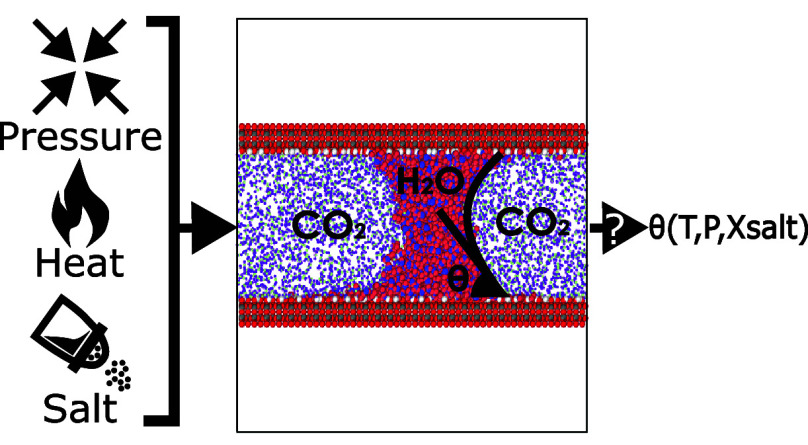

We perform molecular dynamics (MD) simulations of a nanoscale water capillary bridge (WCB) surrounded by carbon dioxide over a wide range of temperatures and pressures (*T* = 280–400 K and carbon dioxide pressures  ≈ 0–80 MPa). The water–carbon dioxide system is confined by two parallel silica-based surfaces (hydroxylated β-cristobalite) separated by *h* = 5 nm. The aim of this work is to study the WCB contact angle (θ_c_) as a function of *T* and . Our simulations indicate that θ_c_ varies weakly with temperature and pressure: Δθ_c_ ≈ 10–20° for  increasing from ≈0 to 80 MPa (*T* = 320 K); Δθ_c_ ≈ −10° for *T* increasing from 320 to 360 K (with a fixed amount of carbon dioxide). Interestingly, at all conditions studied, a thin film of water (1–2 water layers-thick) forms under the carbon dioxide volume. Our MD simulations suggest that this is due to the enhanced ability of water, relative to carbon dioxide, to form hydrogen-bonds with the walls. We also study the effects of adding salt (NaCl) to the WCB and corresponding θ_c_. It is found that at the salt concentrations studied (mole fractions *x*_Na_ = *x*_Cl_ = 3.50, 9.81%), the NaCl forms a large crystallite within the WCB with the ions avoiding the water–carbon dioxide interface and the walls surface. This results in θ_c_ being insensitive to the presence of NaCl.

## Introduction

To achieve net-zero carbon emissions, a broad suite of technologies should be deployed to transform the energy landscape. Carbon capture and storage (CCS) technologies play a pivotal role because they contribute both to reducing emissions in key sectors directly and to removing carbon dioxide (CO_2_) to balance emissions from hard-to-abate industries.^1^ After years of slow progress, CCS is gaining momentum behind new investment incentives and strengthened climate goals.

**Figure 1:**
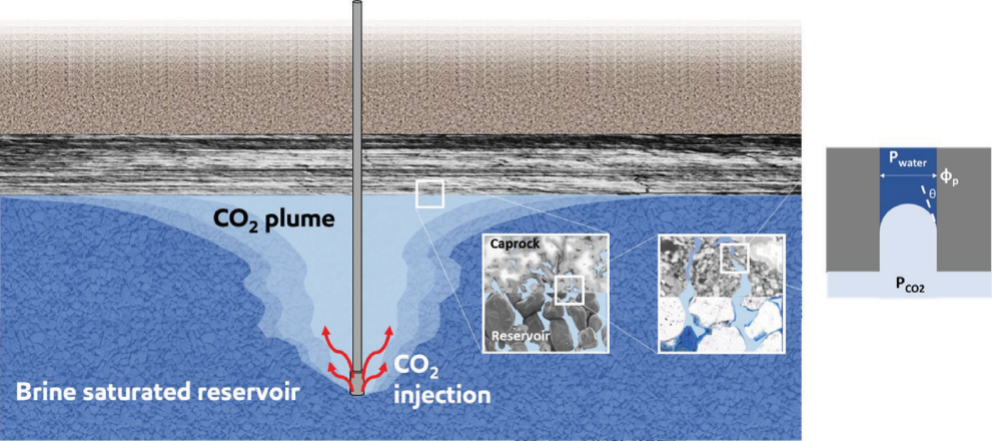
Schematic of CO_2_ structural trapping by a sealing caprock: a buoyant CO_2_ column is held by capillary forces at the caprock. The capillary pressure (eq 1) is a function of wetting properties: interfacial tension σ, contact angle θ, and the pore diameter Φ_P_.

During commercial scale carbon capture and storage operations, CO_2_ is separated and captured from industrial sources and injected deep into and stored in a porous rock formation, such as a depleted hydrocarbon reservoir or saline aquifer. Researchers have proposed other geologic storage options, including in the form of gas hydrates,^2^ CO_2_ storage with enhanced gas recovery,^3^ and enhanced geothermal systems (EGS) using CO_2_ as working fluid.^4^ The target storage formations are greater than 800 m in depth to ensure the injected CO_2_ is in the supercritical state (dense phase), i.e., temperatures >304.25 K and pressure >7.4 MPa (1071 psia).^5^ Conditions for geologic CO_2_ storage typically range between 10–50 MPa in pressure and between 305–393 K in temperature^6^ so CO_2_ remains buoyant because its mass density is about 20% to 50% lower than that of brine. After injection, the buoyancy-driven vertically migrating CO_2_ plume will eventually reach the caprock and be physically held in place by low permeability caprock layers above the storage formation;^7,8^ see Figure 1.

There are four mechanisms that trap CO_2_ in sedimentary rocks.^6^ (i) Dissolution trapping occurs when injected CO_2_ dissolves within the formation brine. (ii) This dissolved CO_2_ can react with rock minerals over long time periods to form carbonate minerals resulting in mineral trapping. (iii) Residual trapping occurs when capillary forces trap “ganglia” of CO_2_ within pore spaces. However, (iv) structural trapping is the primary trapping mechanism for the first few decades after CO_2_ injection, where the caprock acts as a seal both in terms of its low permeability and its high capillary entry pressure.^9,10^

From a macroscopic perspective, the capillary breakthrough pressure *P*_c_ is the maximum pressure difference that exists across the CO_2_–brine interface before CO_2_ percolates across the porous medium^8,11,12^ (Figure 1). The capillary breakthrough pressure can be described using the well-known Young–Laplace equation:
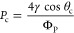
1where γ is the interfacial tension between CO_2_ and brine, θ_c_ is the contact angle of the interface with the mineral surface, and Φ_P_ is the smallest equivalent pore throat diameter along the CO_2_ breakthrough path. CO_2_–brine interfacial tension ranges between 20–30 mN/m for typical CO_2_ storage conditions.^11,13−16^ The contact angle depends on the wetting properties of the caprock minerals in contact with brine and CO_2_ and is used as an indirect method to estimate the effectiveness of the caprock seal. If the caprock minerals are water wet, *P*_c_ is positive, the contact angle is less than 90°, and the pores will retain the buoyant CO_2_.^17,18^ If the caprock minerals are CO_2_ wet, *P*_c_ is negative, the contact angle is greater than 90°, and the CO_2_ is expected to be pulled into pores, potentially leading to leakage.^8^ Contact angle measurements have been reported extensively in the literature on a variety of rock samples including variations in pressure, temperature, and salinity. Most contact angle measurements of water/brine and CO_2_ on quartz and clay substrates report contact angle <50° at temperatures ranging from 296 to 323 K and pressures from 0.1 to 25 MPa.^14,16,19−28^ A few studies report contact angle increase with pressure with most of the change happening at lower pressures (below 10 MPa) and small changes in the supercritical CO_2_ regime.^19,29−31^

Molecular dynamics (MD) simulation studies have covered a broad range of temperatures (298–373 K), pressures (1–20 MPa), and salinities (0–6 M NaCl) and generally predict higher contact angles than experimental measurements. Most studies show the following trends: (i) slight decrease of contact angle (rock becoming more water-wet) with increasing temperature;^32−34^ (ii) increase in contact angle with pressure from 1 to 10 MPa and small changes in the supercritical CO_2_ regime but all mineral surfaces remain strongly water-wet;^17,26,33−35^ (iii) no evidence for a systematic increase or decrease of the contact angle and interfacial tension with salt concentration.^17,33,36^

In this study, we extend previous MD simulation work to temperatures (up to 400 K), pressures (up to 80 MPa), and salinities (up to 9.81 mol %) targeted for geologic CO_2_ storage to address the question as to whether CO_2_ will become the wetting phase instead of brine. This work is organized as follows. We first discuss the computer simulations details. We then present the results where we discuss the effects of temperature, carbon dioxide concentration, and salt (NaCl) on the hydration and contact angle of the water capillary bridge (WCB). We conclude with a brief summary where we discuss the implications of our findings to geological carbon storage.

## Methods

**Figure 2:**
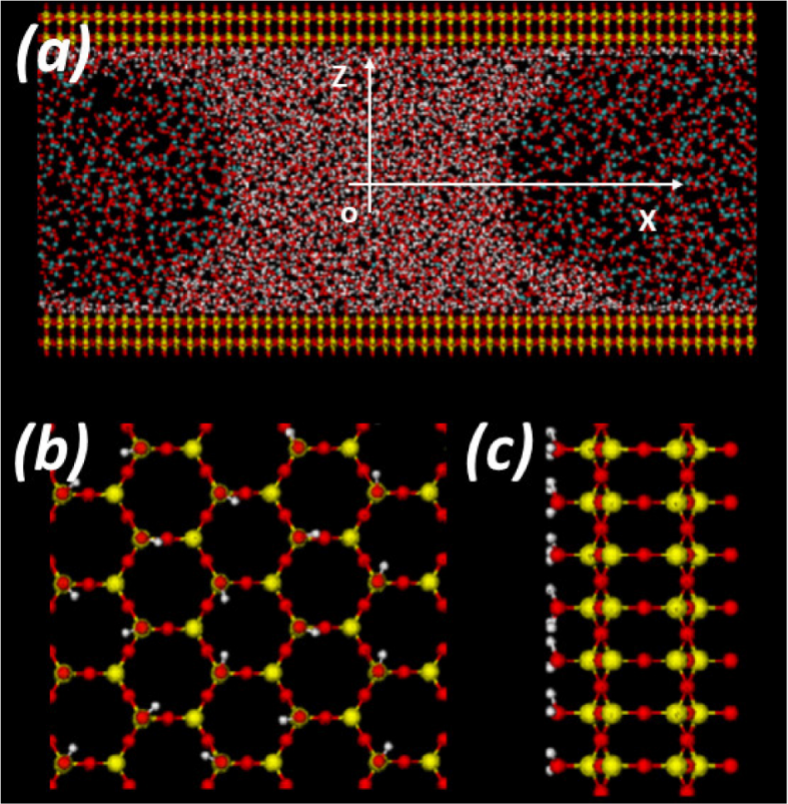
(a) Snapshot of the system from an MD simulation of water (*N* = 2756) and carbon dioxide ( = 1114). Water molecules (center) form a capillary bridge that is surrounded by carbon dioxide molecules [the carbon dioxide molecules also form a capillary bridge (split due to periodic boundary conditions)]. The *z*- and *x*-axis are shown; the origin *o* of the *xz*-reference frame is located at the midpoint between the walls (*z* = 0). (b) Top and (c) side view of a section of the silica walls employed (β-cristobalite). The top view shows the silica tetrahedra forming an hexagonal structure with three silanol groups per hexagon; the silanol groups arrange in a triangular lattice. Only the wall surface in contact with the confined water/carbon dioxide is hydroxylated. The planes containing the H atoms of the walls are located at *z* = ±25 Å and hence, the separation between the walls (defined as the distance between the planes containing the H atoms of each wall) is *h* = 50 Å.

We perform MD simulations of a WCB surrounded by carbon dioxide at *T* = 280–400 K and for (estimated) CO_2_ pressures in the range  = 0–80 MPa. The WCB and carbon dioxide volume are confined by two parallel silica-based (β-cristobalite) walls. A snapshot of one of the systems studied is shown in Figure 2. The WCB is oriented along the *z*-axis, from one wall to the other, and is surrounded by carbon dioxide. The walls extend across the system box along the *x*- and *y*-directions. The system is periodic along the *x*-, *y*-, and *z*-directions with dimensions *L*_*x*_ = *L*_*y*_ = 140.000 Å and *L*_*z*_ = 25.987 Å; the separation between the walls is *h* = 50 Å and a large empty space is left behind the walls in order to minimize any effect from the system periodicity (along the *z*-direction). Given the geometry considered, the WCB that form in our MD simulations are translationally symmetric along the *y*-axis and hence, the WCB profiles depend only on the *z* (WCB height) and *x* coordinates (WCB thickness).

All systems considered are composed of *N* = 2756 water molecules while the number of carbon dioxide molecules varies in the range  = 0–1502 (additional MD simulations are performed using WCB composed of *N* = 1144 water molecules; see Supporting Information). We note that the WCB with *N* = 2756 have a minimum thickness of ≈6 nm and hence, they are sufficiently large so the different (wall–water and water–vapor/CO_2_) interfaces are separated by a volume of bulk-like water molecules that is at least 2–3 nm-thick. As shown in ref.^37^, for capillarity theory/macroscopic thermodynamics to correctly describe the profiles of nanoscale droplets and WCB, the water–wall and water–vapor interfaces should be separated by at least ≈1 nm.^37^ Water molecules are modeled using the SPC/E rigid water model^38^ while the EPM2 flexible model is used to represent the CO_2_ molecules^39^ (see also ref.^40^). The models are validated in the Supporting Information where we compare a few properties obtained from MD simulations and experiments. Each wall is composed of 2352 atoms and is modeled after β-cristobalite. The structure of the walls is described in detail in refs (41−43). Briefly, the walls are composed of silica tetrahedra pointing perpendicularly to the walls surface. The walls are hydroxylated on the confining surface and, hence, both water and carbon dioxide molecules can form hydrogen-bonds (HB) with the walls silanol groups. As in previous studies,^42^ the O and Si atoms of the walls are immobile during the MD simulations. The H atoms of the surface silanol groups are able to rotate in a plane parallel to the wall surface, and about the direction defined by the O–Si covalent bond of the corresponding silanol group.^41,42^

**Figure 3:**
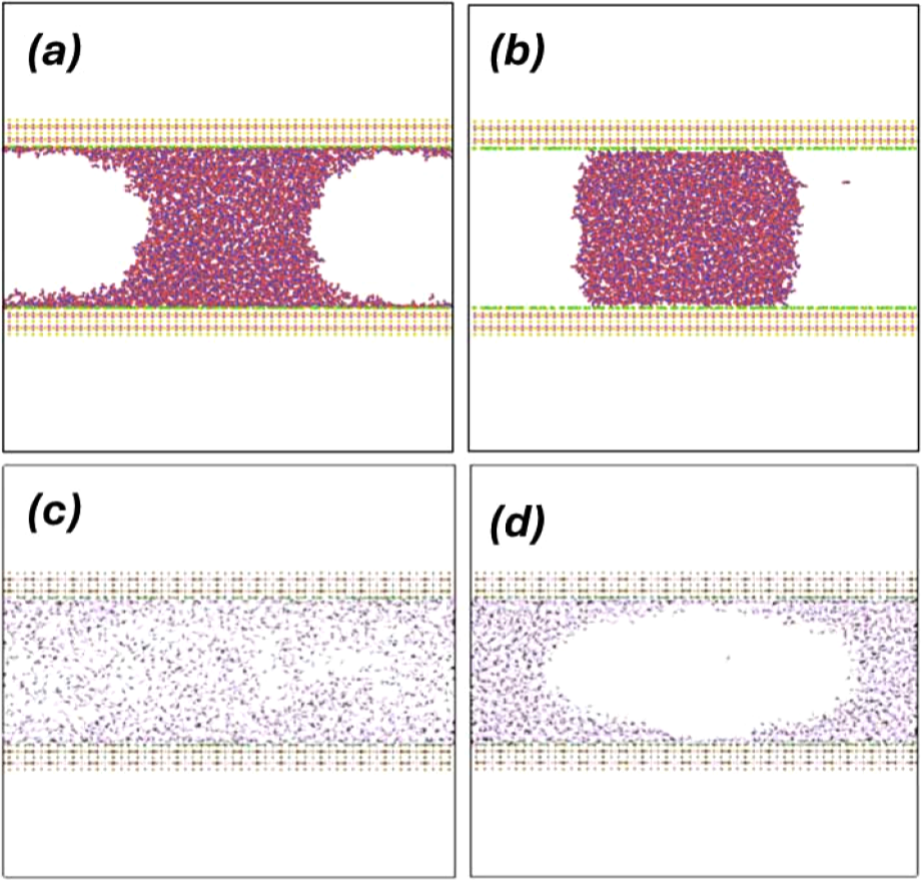
(a) A water capillary bridge formed between two β-cristobalite walls separated by *h* = 50 Å. The walls are hydrophilic and are covered by a thin film of water above which lays the water capillary bridge. (b) Same as (a) but after removing the walls partial charges [when this is done, the surface H atoms (green spheres) have no interactions with water and effectively vanish]. The surfaces are hydrophobic with a water contact angle θ_c_ ≈ 108°. (c), (d) Carbon dioxide confined between two β-cristobalite walls separated by *h* = 50 Å (no water is present;  = 1670). Temperatures are (c) *T* = 320 K (supercritical carbon dioxide), and (d) *T* = 150 K (liquid carbon dioxide). The walls are solvophilic (i.e., they are appealing to the CO_2_ molecules) and are covered by a thin film of carbon dioxide.

We also perform MD simulations of WCB containing salt. In these cases, the WCB contains equal numbers of Na+ and Cl– ions, *N*_Na_ = *N*_Cl_ = *N*_0_ with *N*_0_ = 100, 300 (*N* = 2756). The Na+ and Cl– ions are modeled using the OPLS force field.^44^ As shown in Figure 3a, the surfaces considered are hydrophilic due to the ability of the wall silanol groups to form HB with the water molecules. As shown previously,^42,45^ the water contact angle for the studied surfaces is *θ*_c_ < 10–20°. Accordingly, the water molecules cover the whole wall surface, forming a thin film. The WCB that we study lie on the water films that remain adsorbed at the walls surface. For comparison, we include in Figure 3b the WCB that forms when the wall partial charges (located at the walls silanol groups) are removed, and the H atoms at the wall surfaces effectively vanish (see, e.g., ref.^41^). In this case, there is no water film covering the wall and the contact angle of water is >90°, i.e., the walls become hydrophobic; see also Figure S4.

Our β-cristobalite surfaces are also solvophilic meaning that they are also appealing to CO_2_. As shown in Figure 3c,d, in the absence of water, the CO_2_ molecules cover the wall surface area. Moreover, at low temperatures for which carbon dioxide is stable in the liquid state, a carbon dioxide capillary bridge (CDCB) forms on top of carbon dioxide films adsorbed on the wall surfaces. Indeed, Figure 3d is reminiscent of Figure 3a for the case of water. The solvophilicity of the surface to CO_2_ can be explained by the ability of the surface silanol groups to form HB with the CO_2_ molecules. However, as shown in the Supporting Information, even when the partial charges of the walls (and the H atoms of the corresponding silanol groups) are removed, the walls remain solvophilic to CO_2_ (see Figure S3). It follows that, contrary to the case of water, the walls are appealing to CO_2_ not only due to the formation of wall–CO_2_ hydrogen-bonds but also due to the wall–CO_2_ Lennard-Jones interactions (see Supporting Information).

All MD simulations are performed using the LAMMPS software package.^46^ Simulations are performed for 20 ns; the first 10 ns are used for equilibration and the remaining 10 ns are used for data analysis. The simulation time step is d*t* = 0.001 ps. Our simulations seem to be long enough for the WCB to reach equilibrium; however, we cannot exclude the possibility that the WCB studied here (and in other computational studies) remain metastable during the simulated time – a limitation inherent to all MD simulations.^47−50^ MD simulations are performed at constant volume and temperature; the temperature is maintained using a Nosé–Hoover style thermostat with a coupling time constant of 0.1 ps. Electrostatic interactions are calculated using the particle–particle particle–mesh solver^51^ with a cut off distance of *r*_cutoff_ = 10.0 Å. The same cut off distance is used to calculate the Lennard-Jones (LJ) interactions. Since we employ the same computational techniques used in our previous studies, we refer the reader to refs (42,50) for additional details.

### Calculation of the Capillary Bridge Profiles

The WCB profiles are calculated from 2000 snapshots taken every 5 ps during the last 10 ns of the simulation. The procedure to calculate the WCB is described in detail in ref.^42^. Briefly, for each snapshot, we first define a *z*-axis passing through the center of mass of the WCB, perpendicular to the walls. The WCB is then covered with 20 overlapping slabs of thickness 5 Å parallel to the walls and shifted vertically by 2.5 Å with respect to each other. For each slab, centered at a distance *z* from the midpoint between the walls , we calculate the average density of water ρ_slab*-z*_(*x*) as a function of the distance *x* (WCB thickness) from the *z*-axis [Figure 2a]. As expected, ρ_slab*-z*_(*x*) is constant within the WCB and it decays abruptly to practically zero in the vapor phase or CO_2_ volume. Hence, we define the thickness of the WCB at height *z*, *x*_MD_(*z*), as the distance *x* at which ρ_slab*-z*_(*x*) = ρ_0_ = 0.2 g/cm^3^ (our results are not sensitive to slight variations in ρ_0_). The function *x*_MD_(*z*) provides the WCB profile for the given snapshot. By averaging *x*_MD_(*z*) over the 2000 snapshots considered, we obtain the average WCB profile.

The procedure described so far to obtain *x*_MD_(*z*) applies to an isolated WCB and to a WCB surrounded by CO_2_ molecules – in the presence of carbon dioxide, only a small number of CO_2_ molecules are able to diffuse within the WCB (see below). However, the situation is different when salt is present since Na+ and Cl– ions remain within the WCB. Accordingly, when the WCB contains NaCl, we do not calculate the *density* profile ρ_slab*-z*_(*x*) but, instead, we obtain the number density *n*_slab*-z*_(*x*) of the water O, as well as the Na+ and Cl- ions. Specifically, in the calculation of *n*_slab*-z*_(*x*), the water O, Na+, and Cl– ions all are treated identically as a “particle”. As expected, *n*_slab*-z*_(*x*) is constant within the WCB and it decays abruptly to zero in the vapor phase or CO_2_ volume. Hence, we define the thickness of the WCB (containing NaCl) at height *z*, *x*_MD_(*z*), as the distance *x* at which *n*_slab*-z*_(*x*) decays to *n*_0_ = 6.685 nm^–3^. This choice for the value of *n*_0_ is roughly equivalent to the condition ρ_slab_*_-z_*(*x*) = ρ_0_ = 0.2 g/cm^3^ used to define *x*_MD_(*z*) in the case of WCB containing no salt.

### Calculation of the Water Contact Angle from the Water Capillary Bridge Profile

Capillarity theory predicts that the profile of a translationally symmetric capillary bridge, such as the WCB formed in our MD simulations, should be a circle of radius *R*_2_ and centered at (*x*_c_,*z*_c_), where *z*_c_ = 0 and *x*_c_ = *r*_0_ – *R*_2_ (*r*_0_ is half the thickness of the capillary bridge at *z* = 0); see ref. (42). Accordingly, to calculate the contact angle of water from the WCB obtained in our simulations, we fit the corresponding average WCB profile *x*_MD_(*z*) with a circle:
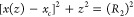
2The function *x*(*z*) that best fits the WCB profile *x*_MD_(*z*) using eq 2 can be used to calculate the contact angle of water. Specifically, we get θ_*c*_ from the function *x*(*z*) using the expression  at *z* = *z*_0_. In principle, one would need to evaluate this expression at the wall surface, i.e., *z*_0_ = *h*/2. However, as shown below, the walls studied are always covered by a film of water which makes it unclear how to define the height *z*_0_ at which the WCB ends and merges with the water film adsorbed at the wall surface. In our simulations, to avoid any artifact due to the water films on the walls, we only fit the average WCB profile *x*_MD_(*z*) for |*z*| < 17.5 Å, and estimate θ_c_ using *z*_0_ = 17 Å. For comparison, we also estimate θ_c_ using the same procedure described above but, instead of using eq 2, we fit the WCB profile with a second order polynomial.

## Results and Discussion

The results are organized as follows. We first discuss the walls hydration and the properties of the WCB in the presence of carbon dioxide at different temperatures and pressures. We then discuss the effects of adding salt (NaCl) on the wetting and WCB properties.

### Water Capillary Bridges in the Presence of Carbon Dioxide

#### Wall Hydration

**Figure 4:**
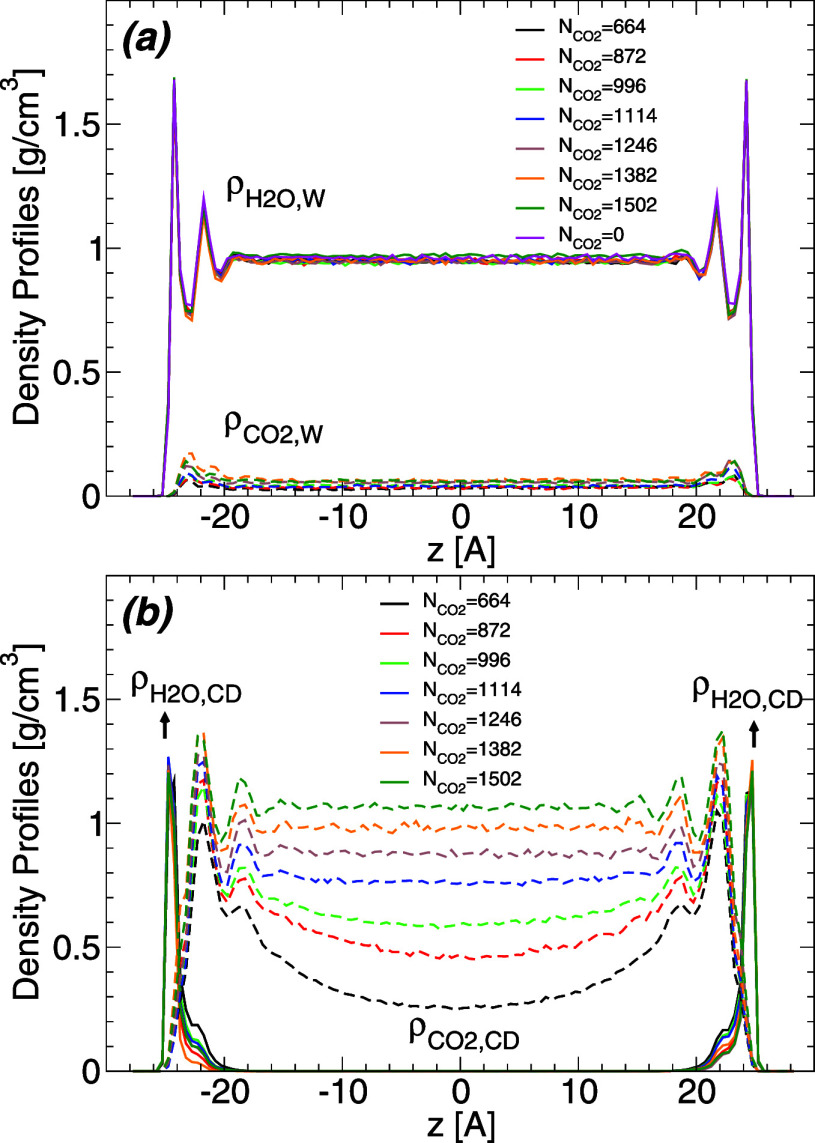
(a) Density profile of water (upper solid lines; ) and carbon dioxide (lower dashed lines; ) within the water capillary bridge, and along the direction perpendicular to the walls. Results are for *T* = 320 K and different number of carbon dioxide molecules .  is practically independent of the amount of carbon dioxide in the system; the two maxima next to the walls (located at *z* = ± 25 Å) indicate that two well-defined water layers form next to each wall.  is small, i.e., the amount of CO_2_ within the WCB is minor at all conditions studied. The walls under the WCB are solvated preferentially by water. (b) Density profile of water (solid lines; ) and carbon dioxide (dashed lines; ) within the carbon-dioxide capillary bridge (CDCB), and along the direction perpendicular to the walls; *T* = 320 K. The maxima of  indicate that water molecules preferentially hydrate the walls (forming a thin film of water separating the carbon dioxide volume and the walls). Not surprisingly,  varies considerably with the amount of carbon dioxide in the system, with densities from 0.25 g/cm^3^ (gas) to ≈1.1 g/cm^3^ (liquid) at the midpoint between the walls (*z* = 0).

In order to characterize the hydration of the walls, we first calculate the water and carbon dioxide density profiles *within the WCB* and along the direction perpendicular to the walls (*z*-direction),  and . To do so, we calculate the center of mass of the WCB and consider only those H_2_O/CO_2_ molecules within a distance Δ*x* = 20 Å along the *x*-axis from the WCB center of mass. The value of Δ*x* is small enough to exclude any artifact due to the water–carbon dioxide interface. Figure 4a shows  and  at *T* = 320 K and for different amounts of carbon dioxide. At these conditions, carbon dioxide is supercritical since the critical temperature of the flexible EPM2 carbon dioxide model is *T*_c_ ≈ 313 K.^39^ Nonetheless, similar results are obtained at *T* = 280–400 K. Figure 4a shows that  is practically independent of the presence of CO_2_. In particular,  exhibits two maxima next to the walls, at approximately |*z*| = 24.5 Å and |*z*| = 22.0 Å, indicating the formation of two well-defined hydration layers next to the walls. At distances ≈8–10 Å away from the wall surfaces, the density of water within the WCB is constant and approximately equal to the density of bulk SPC/E water at *T* = 320 K and low pressures.^52^ As shown in Figure 4a,  is rather small for all values of *z* indicating that only a few CO_2_ molecules are able to diffuse into the WCB (the mass fraction of carbon dioxide is <10%). The few CO_2_ molecules within the WCB tend to locate preferentially at approximately |*z*| = 23 Å, in between water first and second hydration layers. Therefore, under the WCB, the walls are preferentially solvated by water, as expected.

We also calculate the water and carbon dioxide density profiles *within the carbon dioxide capillary bridge (CDCB)* and along the direction perpendicular to the walls (*z*-direction),  and . To do so, we first calculate the center of mass of the CDCB, and consider only those molecules within a distance Δ*x* = 20 Å from the CDCB center of mass. Again, the value of Δ*x* is small enough to exclude any artifact due to the water–carbon dioxide interface. Figure 4b shows  and  at *T* = 320 K for different amounts of carbon dioxide (similar results are obtained at *T* = 280–400 K).  shows a single peak at |*z*| = 24.5 Å, practically independent of the presence of CO_2_, indicating the formation of a thin film of water adsorbed at the wall surface. Interestingly,  ≈ 0 for −19 < *x* < 19 Å meaning that water molecules do not diffuse into the carbon dioxide volume. The CO_2_ molecules form 1–2 layers close to the walls. The first maximum of  is located at |*z*| = 23 Å and, hence, the carbon dioxide volume is separated from the walls by approximately one layer of water molecules.

**Figure 5:**
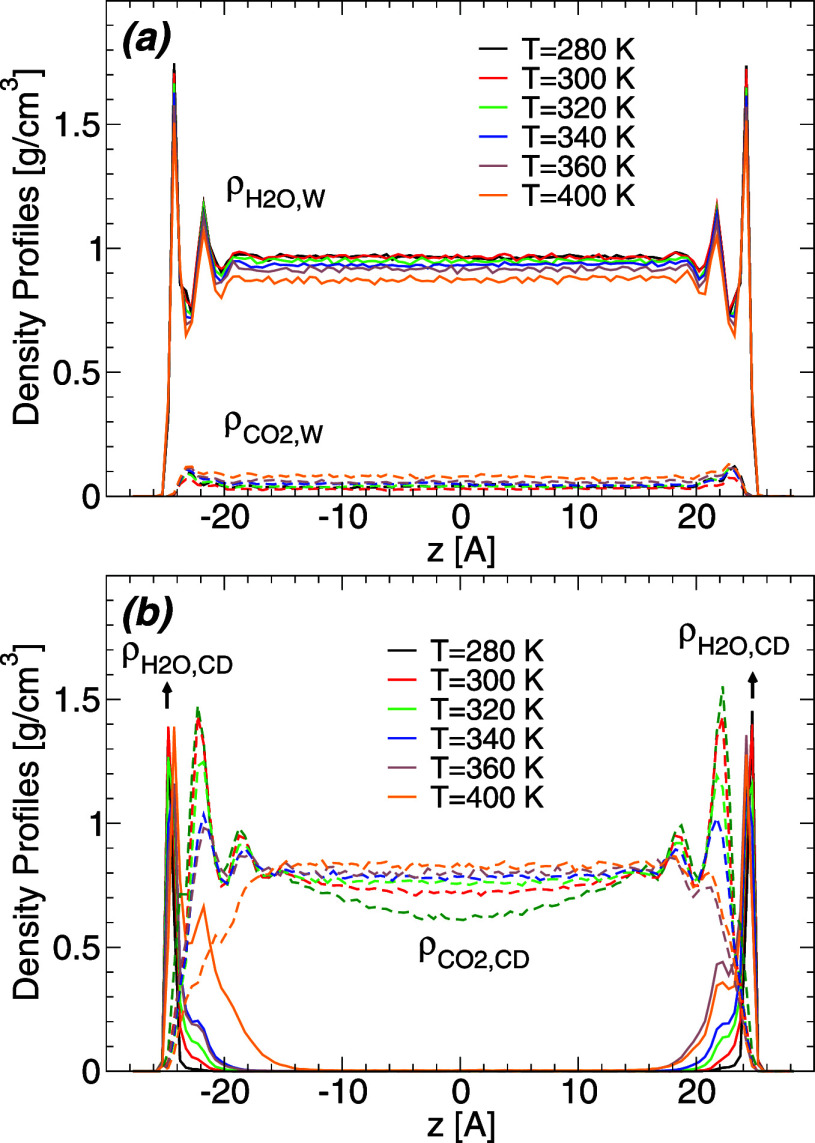
(a) Temperature effects on the density profile of water (upper solid lines; ) and carbon dioxide (lower dashed lines; ) within the water capillary bridge, and along the direction perpendicular to the walls. Results are for  = 1114 and *T* = 280, 300, 320, 340, 360, 400 K (the critical temperature of carbon dioxide for the model studied is *T*_c_ ≈ 313 K). Varying the temperature causes slight changes in the local density of water and carbon dioxide within the WCB but it does not affect the location of the water/carbon dioxide layers (maxima in  and ) close to the walls. (b) Density profile of water (solid lines; ) and carbon dioxide (dashed lines; ) within the CDCB, and along the direction perpendicular to the walls. Temperature variations affect slightly the local density of water and carbon dioxide within the CDCB, increasing the number of water molecules in the water films adsorbed at the walls surfaces and pushing the CO_2_ molecules away from the walls [toward the central region of the CDCB (*z* = 0)].

The results discussed so far are for *T* = 320 K but qualitatively similar results are found at *T* = 280, 300, 340, 360, and 400 K. To show this, we include in Figure 5a the  and  obtained for  = 1114 and at all temperatures studied (results for  = 1502 are included in the Supporting Information). The main effect of increasing the temperature, for a fixed , is to decrease the height of the maxima in  close to the walls and the density of the “bulk” water away from the walls (i.e., at −19 < *x* < 19 Å). The decrease in the water density within the WCB leads to an increase in  for all values of *z*. In other words, more CO_2_ molecules are able to diffuse into the WCB with increasing temperature, implying that the solubility of CO_2_ in water increases upon heating. This may seem inconsistent with experiments which show that the solubility of CO_2_ decreases upon heating.^53^ However, experiments are performed at constant pressure while our MD simulation are performed at constant volume, and fixed amounts of water and carbon dioxide. Nonetheless, the change of the carbon dioxide density within the WCB is small,  < 0.10–0.12 g/cm^3^ for −20 < *x* < 20 Å, so the number of CO_2_ molecules within the WCB remains low at all temperatures studied. We also note that the locations of the water and carbon dioxide layers within the WCB, in the proximity of the walls, do not change with temperature.

The temperature effects on the density profiles of water and carbon dioxide within the CDCB are shown in Figure 5b. Included are the  and  obtained for  = 1114 at all temperatures studied (results for  = 1502 are included in the Supporting Information). The main effect of increasing the temperature, for a fixed value of , is to thicken the water layer separating the carbon dioxide volume and the walls. At the highest temperature studied, *T* = 400 K, the water film on the walls seems to be composed of two water layers while there is practically a single water layer at *T* < 400 K. The thickening of the water films next to the walls, with increasing temperature, leads to a reduction of the volume available to the carbon dioxide. Accordingly, as shown in Figure 5b, (i)  decreases in the proximity of the walls as the temperature increases while (ii) it increases upon heating at  Å (corresponding to the “bulk” carbon dioxide volume within the CDCB). Briefly, the CO_2_ molecules are pushed away from the wall as the temperature increases.

**Figure 6:**
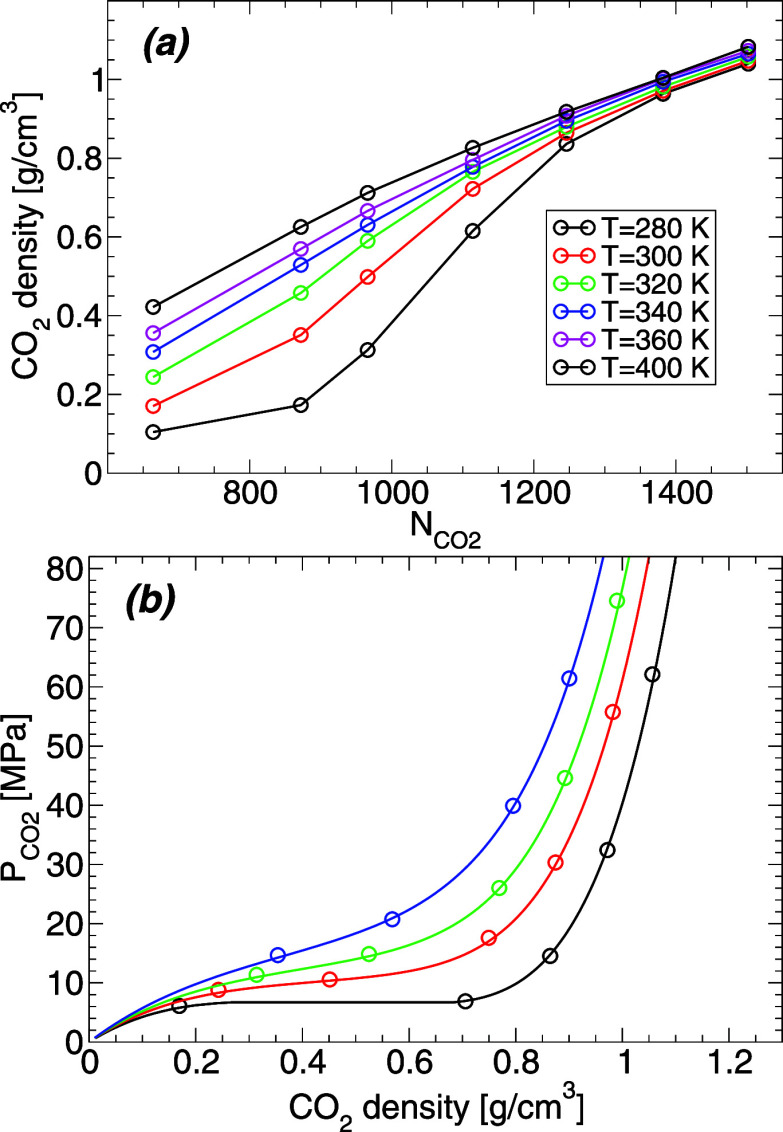
(a) Density of carbon dioxide within the CDCB (defined by the value of  at *z* ≈ 0) for  = 664, 872, 966, 1114, 1246, 1382, 1502 and all temperatures studied. (b) Estimated carbon dioxide pressure, , as a function of density within the CDCB (*T* = 280, 300, 320, 340 K, bottom to top). Lines are the (ρ)-equation of state from experiments of bulk carbon dioxide; circles are the pressures corresponding to the densities included in (a) along a given isotherm.

The results presented so far are given in terms of . However, it is not practically feasible to measure ; experiments usually have access to the pressure of carbon dioxide, . We can estimate  in our MD simulations [at a given (*T*, )] from the value of  at *z* ≈ 0. Specifically, the carbon dioxide within the CDCB at *z* ≈ 0 is far from the walls and hence, it may be considered to have carbon dioxide bulk-like properties. Hence, the pressure within the carbon dioxide in our simulations can be estimated by the pressure of bulk CO_2_ at a density equal to the value of  at *z* ≈ 0. We stress that the pressure–density equation-of-state for bulk CO_2_ obtained from experiments and MD simulations (flexible EPM2 model) are in very good agreement; see Figure S1. Therefore, we estimate the pressure of carbon dioxide within the CDCB as the *experimental* pressure of bulk carbon dioxide at the density  at *z* ≈ 0 (at the temperature considered). Figure 6a shows  as a function of  obtained by following the procedure described above. The values of  as a function of the CO_2_ density, as defined by  at *z* ≈ 0, is shown in Figure 6b. It follows from Figure 6 that carbon dioxide is at supercritical conditions at *T* ≥ 320 K, with carbon dioxide being in a liquid-like state for  = 1382 and 1502 (the highest two density values along an isotherm), and gas-like state for  = 664 and 872 (the lowest two density values along an isotherm).

#### Water Capillary Bridge Profile and Contact Angle

**Figure 7:**
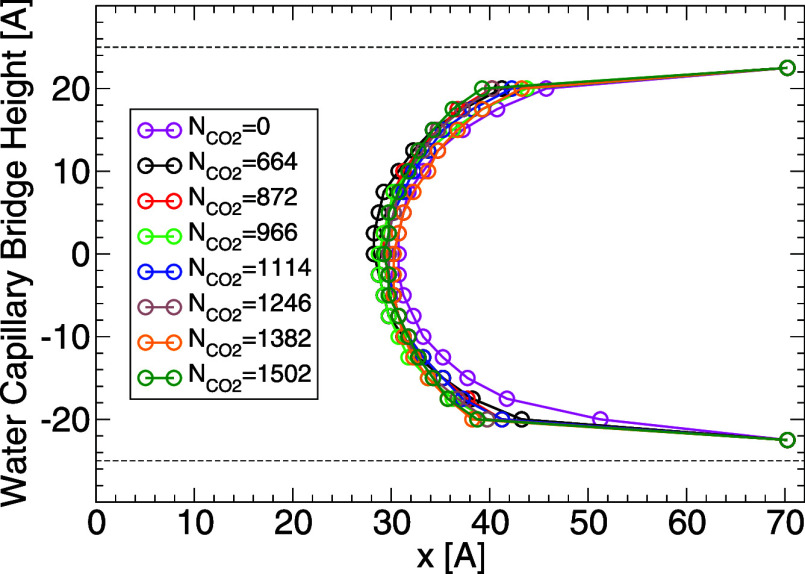
Profile of the WCB at *T* = 320 K and for different amounts of carbon dioxide. Similar results are obtained at the other temperatures studied. The dashed lines at *z* = ±25 Å indicate the location of the walls surface (defined by the planes containing the H atoms of the walls). The data points at heights *z* = ±22.5 Å are off due to the water film adsorbed on the wall surfaces and hence, these data points do not represent the WCB profile.

In order to explore the effects of carbon dioxide on the wetting behavior of the WCB, we focus on the case of *T* = 320 K (at which carbon dioxide is supercritical). Similar results are obtained at the other temperatures studied. Figure 7 shows the average WCB profile in the presence of  = 0, 664, 872, 966, 1114, 1246, 1382, and 1502 carbon dioxide molecules. As shown in Figure 6b (red line, *T* = 320 K), the systems studied correspond to CO_2_ pressures in the range  ≈ 0–80 MPa. It follows from Figure 7 that the WCB profiles are weakly affected by variations in the amount of carbon dioxide.

**Figure 8:**
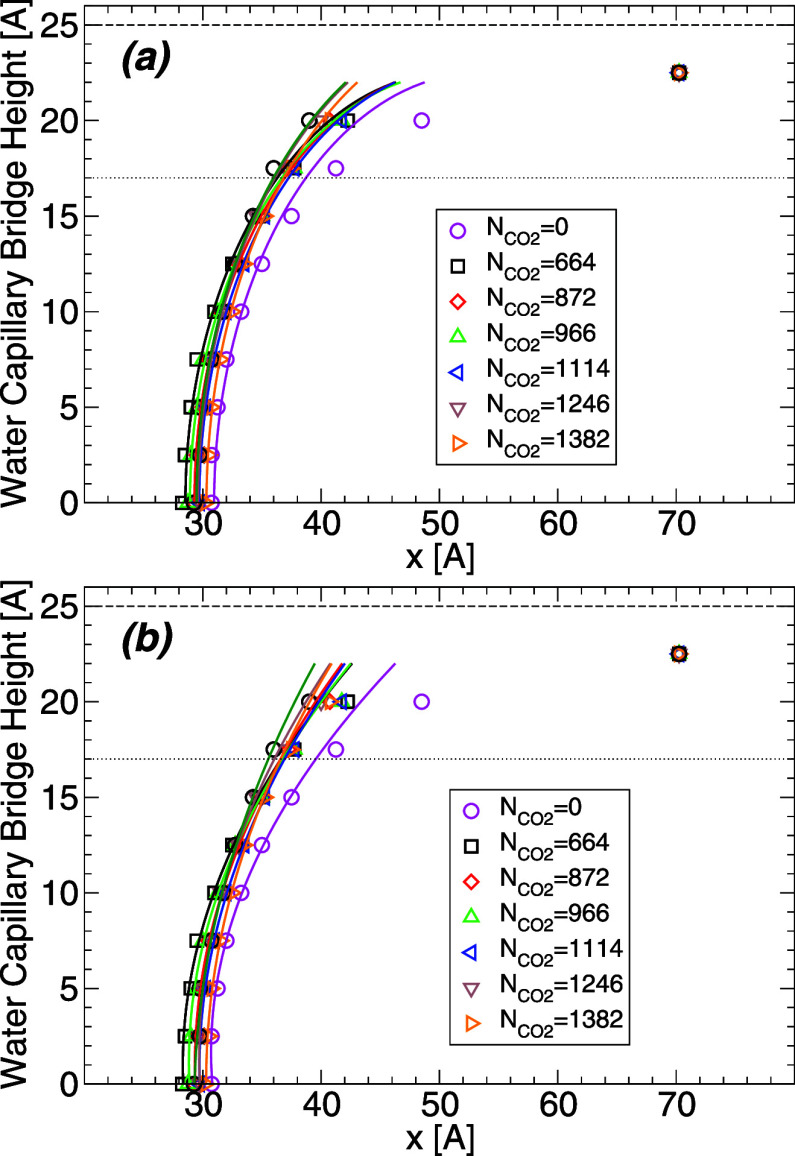
(a) Upper half of the WCB shown in Figure 7 at *T* = 320 K and for different amounts of carbon dioxide (symbols). Lines are obtained by using a circle to fit the WCB profile (eq 2). (b) Same as (a) with the lines representing a second order polynomial fit of the WCB profiles. Only data points at *z* < 17.5 Å are used in the fitting procedure in order to exclude any contribution from the water films adsorbed on the walls. The wall surface is located at height *z* = 25 Å (dashed line); the dotted line corresponds to *z* = 17 Å at which the contact angle is evaluated (Figure 9).

**Figure 9:**
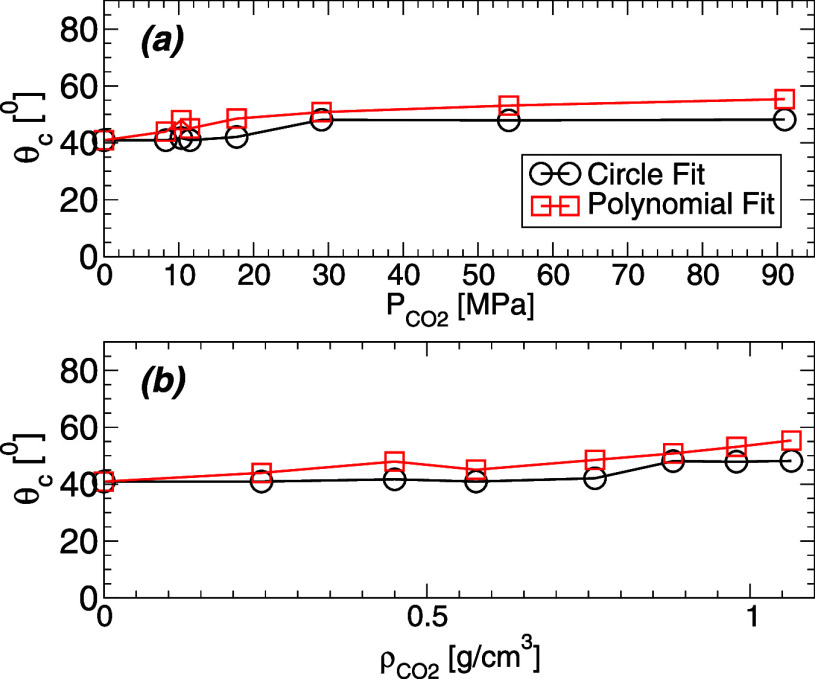
Water contact angle θ_c_ at *T* = 320 K obtained from the WCB shown in Figure 8. (a) θ_c_ as a function of the carbon dioxide pressure,  (see Figure 6). (b) θ_c_ as a function of the carbon dioxide density, . Circles are the θ_c_(*T*) resulting from the fits of the WCB in Figure 8a using a circle [eq 2]. Squares are the θ_c_(*T*) resulting from the fits of the WCB in Figure 8b using a second order polynomial. In both cases, θ_c_(*T*) increases weakly (Δθ_c_ ≈ 10–20°) as the amount of carbon dioxide increases. Estimated error bars are approximately 2–4° (smaller than the symbols size).

To estimate the contact angle of water, we fit the WCB profiles shown in Figure 7 with either a circle, or a second order polynomial. The corresponding fits to the WCB profiles are shown in Figure 8. Both fits work reasonably well at heights |*z*|< 17 Å, corresponding to distances of at least 8 Å away from the nearest wall. The contact angles of water obtained from both fitting procedures are shown in Figure 9. Our MD simulations indicate that θ_c_ ≈ 40–60° depending on the conditions and the method considered. These values are not inconsistent with experiments (where θ ≈ 0–60° depending on the surface and experimental details^54^) and are slightly larger than the contact angles of WCB reported from computer simulations using different model surfaces and methods (e.g., θ_c_ ≈ 0–45° in refs (40,54)). The main point of Figure 9 is that, independently of the method used to calculate the WCB contact angle, our MD simulations indicate that θ_c_ increases weakly with increasing the carbon dioxide density/pressure, Δθ_c_ ≈ 10–20° for  = 0–80 MPa (and *T* = 320 K).

Our choice of (A) fitting the WCB profiles up to |*z*| < *z*_c_ = 17.5 Å, and (B) evaluating θ_c_ from the slope of the so-obtained WCB profile at *z*_0_ = 17.0 Å is based on physical grounds. (i) Macroscopic thermodynamics (capillarity theory) assumes that the WCB is composed of a bulk water volume bounded by the water–wall and water–vapor (or water–CO_2_) interfaces. However, as shown in Figures 4a and 5a, two layers of water molecules form next to the walls and  becomes constant only at a distance approximately *d* > 8–10 Å from the walls. Accordingly, from a capillarity theory point of view, the “bulk” water within the WCB extends up to, at most, the distance *d* from the walls and hence, evaluation of the WCB profile should exclude those molecules located at |*z*| > (25 Å – *d*) (wall–water interfaces). (ii) We also note that in a previous study,^37^ we tested the ability of capillarity theory to predict the profile of WCB confined by the same silica walls employed here but at smaller wall separations, *h* < 50 Å. It was found that capillarity theory correctly predicted the WCB profile down to approximately *h* ≈ 25–30 Å. At smaller values of *h*, capillarity theory failed; at these separations there is not bulk-like water between the walls (the two water-wall interfaces practically touched each other). Briefly, the results of ref. (37) also indicate that, macroscopically, it is not physically consistent to include the water molecules (films) next to the wall (within a distance ≈*d*) when applying capillarity theory (e.g., to fit the WCB profile and measure the associated contact angle). To do so would require modifying capillarity theory, see ref. (37). (iii) In addition, the film of water on the wall and under the CO_2_ volume extends up to ≈7–8 Å from the wall [see the density profile  in Figures 4b and 5b]. This means that a reliable WCB profile can only be calculated for approximately |*z*| < 17–18 Å. Otherwise, when one calculates the radius of the WCB at a given position *z*, molecules from the water film may be (erroneously) included in the calculations; see the Methods section.

##### The Role of Temperature

**Figure 10:**
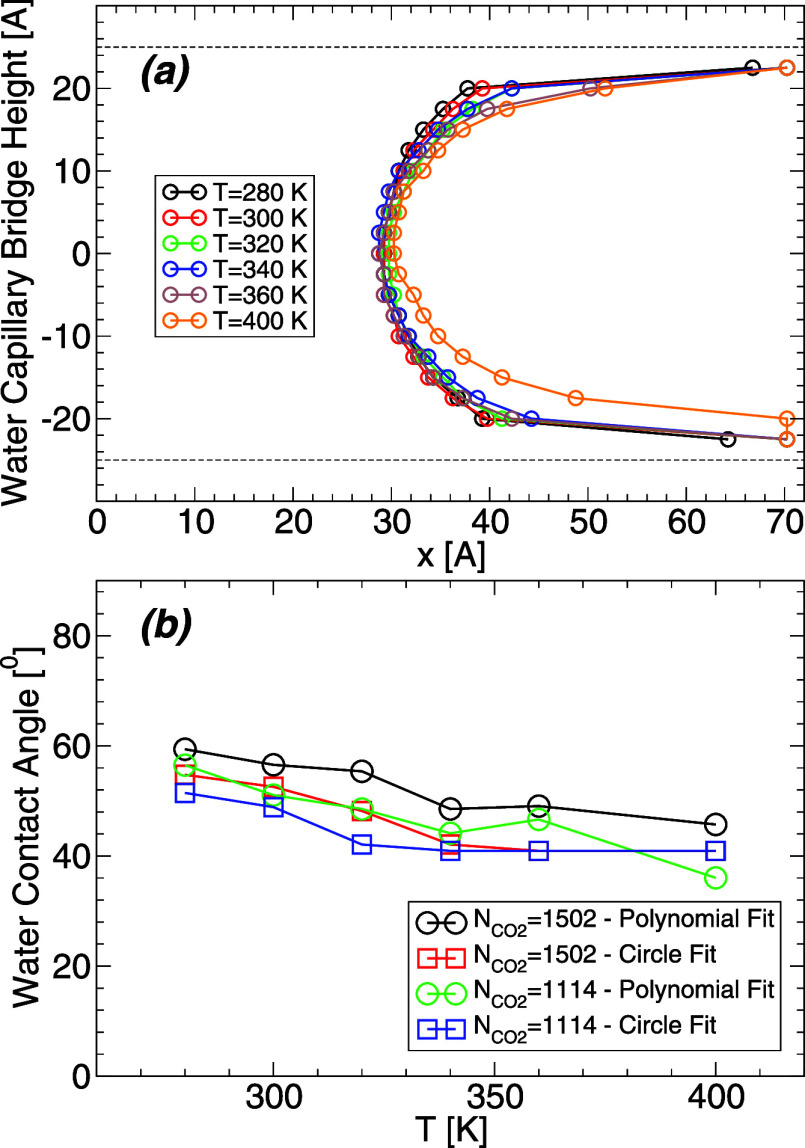
(a) Profile of the WCB surrounded by carbon dioxide, , at different temperatures. Similar results are obtained for other values of . The dashed lines indicate the location of the walls surface (as defined by the plane containing the H atoms of the wall). The WCB profiles for heights *z* = ±22.5 Å are off due to the presence of water films adsorbed on the walls; these data points do not represent the WCB profile. (b) Water contact angle θ_c_ obtained from (a) for  (green and blue lines). Also included, are the results for  = 1502 (black and red lines). Estimated error bars are approximately 2–4° (smaller than the symbols size).

Next, we focus on the role of temperature on the WCB profile and water contact angle for a fixed amount of carbon dioxide. The WCB profiles surrounded by  = 1114 carbon dioxide molecules at *T* = 280–400 K are shown in Figure 10a. Figure 10b shows the values of θ_c_ obtained from Figure 10a when the WCB profiles are fitted by a circle or a quadratic polynomial (green and blue lines). Despite the noise in the data, our results suggest that θ_c_ decreases slightly with increasing temperature. For example, θ_c_ decreases by <10° when the temperature increases from *T* = 320 to 360 K.

#### Why Does Water Form a Film between the Walls and Carbon Dioxide?

**Figure 11:**
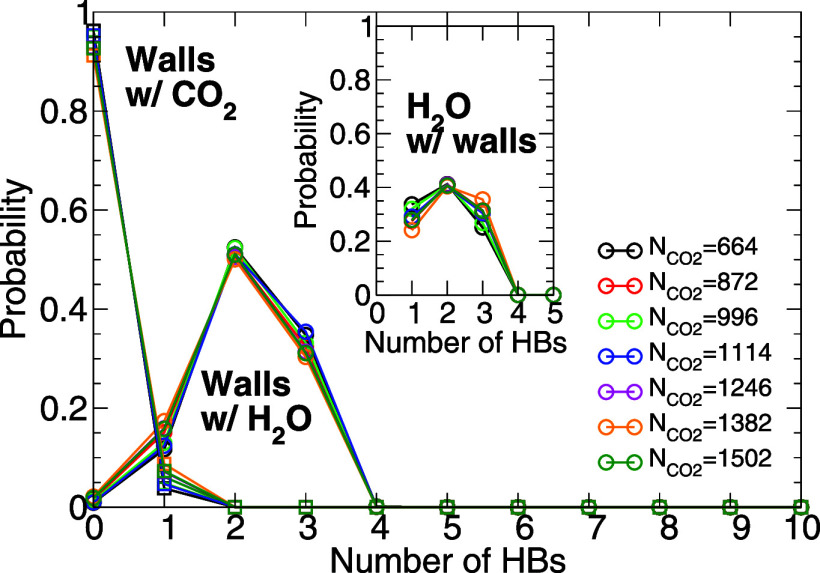
Probability distribution for the number of HB that a silanol group of the walls forms with H_2_O and CO_2_ molecules. Only silanol groups located under the CDCB are considered. Most of these silanol groups form no HB with the CO_2_ molecules and only ≈5% are able to form one HB with the CO_2_ molecules. Instead, these silanol groups can form up to 3 HB with H_2_O molecules. Inset: probability distribution for the number of HB that a H_2_O molecule forms with the wall silanol groups. In this case, only molecules that form at least one HB are included in the statistics. Results are based on a system composed of *N* = 2756 and  =1114 molecules at *T* = 320 K.

To answer this question, we calculate the number of HB that the walls silanol groups form with H_2_O/CO_2_ molecules. The main panel of Figure 11 shows the probability distribution for the number of HB, *P*(*n*_HB_), that a silanol group forms with nearby (i) H_2_O and (ii) CO_2_ molecules. In the case of CO_2_, the distribution *P*(*n*_HB_) > 0 only for *n*_HB_ = 0, 1 meaning that the silanol groups can form at most 1 HB with CO_2_ molecules. Indeed, we find that most silanol groups do not form HB with CO_2_ molecules and only ≈5% of the surface OH groups form one HB with the CO_2_ molecules. Instead, for the case of water, *P*(*n*_HB_) > 0 for *n*_HB_ = 1, 2, 3, i.e., the surface OH groups can form up to 3 HB with water molecules. About 50% and 35% of the OH groups form 2 and 3 HB with H_2_O molecules, respectively.

**Figure 12:**
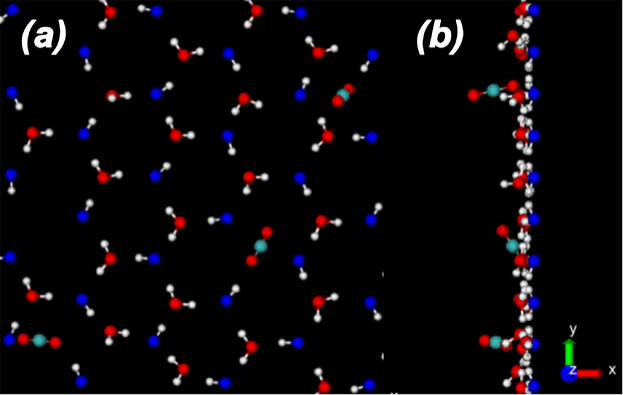
(a) Top and (b) side view of a section of one of the walls in contact with the carbon dioxide capillary bridge. Only the wall surface O and H atoms are shown (blue and white spheres, respectively). The wall surface OH groups are located in a triangular lattice [see also Figure 2b]. Also included are the (approximately 20) water and (three) carbon dioxide molecules that form HB with the surface OH groups. These CO_2_ molecules form one HB with the wall OH groups and they tend to stick away from the wall. The H_2_O molecules form mostly 2–3 HB with the OH groups by sitting flat and parallel to the walls, in between three of the surface OH groups [H_2_O molecules sit at the center of the triangular lattice formed by the OH groups or, equivalently, they sit at the center of the hexagonal lattice formed by the silica tetrahedra in Figure 2b].

We also find that a given water molecule can form multiple HB with the walls silanol groups. To show this, we consider only those water molecules that form at least one HB with the walls and evaluate the total number of HB that they form with the surface silanol groups. As shown in the inset of Figure 11, such water molecules can form 1, 2, and even 3 HB with the silanol groups. To confirm these results, we include in Figure 12 a typical snapshot of the system showing only those H_2_O and CO_2_ molecules that form at least one HB with the walls OH groups. Water molecules are able to occupy the spaces between three silanol groups (at the center of the hexagons shown in Figure 2b), while forming up to three HB with the surface OH groups. Instead, the CO_2_ molecules tend to stick away from the walls and form only one HB with the surface OH groups. Clearly, in the case of β-cristobalite, the topography of the surface, and the distribution of silanol groups, are important factors that favor the formation of HB between water and the walls, relative to carbon dioxide. Similar results are expected for the case of other surfaces composed of silica tetrahedra, such as α-quartz. For example, in ref.^55^ it is found that water molecules can make 2–3 HB with silanol groups on the surface of silicalite-1, a widely studied zeolite. However, from a chemistry point of view, one may expect that, in general, water will preferentially wet the surface if the surface has the ability to form HB with H_2_O/CO_2_. This is because a CO_2_ molecule can only “accept” HB with its O atoms while, instead, a H_2_O molecule can “accept” two HB, with its O atom, and “donate” two HB, with its two H atoms.

Summarizing, the walls are preferentially solvated by water because of the ability of the H_2_O molecules to form more HB (per molecule) than the CO_2_ molecules. This allows the system to lower the enthalpy and the free energy (assuming the entropic contribution plays a secondary role).

### The Role of Salt (NaCl) on the Wall Hydration, Water Capillary Bridges, and Water Contact Angle

#### Walls Hydration

**Figure 13:**
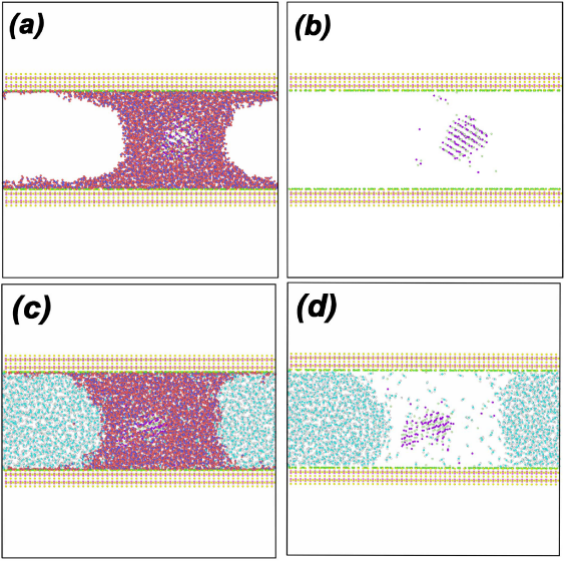
(a) Water capillary bridge containing *N*_0_ = 100 pairs of Na+ and Cl− ions at *T* = 320 K (*N* = 2756 water molecules). (b) Na+ and Cl– ions within the WCB shown in (a). The ions remain within the WCB at all times and stay away from the walls and water–carbon dioxide interface. At the present concentration (*x*_Cl_ = *x*_Na_ = 100/(100 + 2756) = 3.50% mole fraction), the ions form a crystallite inside the WCB. The rod-like crystallite extends through the WCB bridge length (*y*-direction), and is effectively infinite due to periodic boundary conditions. (c), (d) Same as (a), (b) for a WCB in the presence of carbon dioxide (*T* = 320 K, *N* = 2756,  = 1502, *N*_0_ = 100).

Figure 13a shows a snapshot from our MD simulations of a WCB containing *N*_0_ = 100 pairs of Na+ and Cl– at *T* = 320 K, with no carbon dioxide. Our simulations show that the ions aggregate and form a crystallite that remains within the WCB; see Figure 13b. Hence, the Cl– and Na+ ions remain preferentially away from the water–vapor interface and do not diffuse into the empty space. Interestingly, the Cl– and Na+ ions also remain away from the water–wall interface. Similar results are found for the case of *N*_0_ = 300 pairs of Na+ and Cl–. It is not clear whether the tendency of NaCl to form crystallites within the WCB is due to (i) confinement (nm-scale WCB and nm-scale wall–wall separation), or to (ii) the Na/Cl/water interactions, or both. In the Supporting Information, we present results from MD simulations of NaCl in bulk water (*T* = 300 K, *p* = 0.1 MPa) and mole fractions *x*_Cl_ = *x*_Na_ = 0.91, 1.80, 4.93, 9.40%. Crystallites are found for *x*_Cl_ = *x*_Na_ = 4.93, 9.40% but not for *x*_Cl_ = *x*_Na_ = 0.91, 1.80%. The effective concentration of ions within our WCB for *N*_0_ = 100 should be, at least, *x*_Cl_ = *x*_Na_ = 100/(100 + 2756) = 3.50% since many water molecules belong to the films adsorbed on the walls. Accordingly, it seems plausible that the ions form a crystallite within the WCB mostly due to the Na–Cl–water interactions, with confinement effects playing a secondary role (note that at *x*_Cl_ = *x*_Na_ = 4.93, 9.40%, we would need *N*_0_ 175, 350 pairs of Na+ and Cl– ions in the WCB). We note that the force field used in our computational study (and other studies) has its own limitations. For example, the concentrations at which we find the formation of crystallites is smaller than the experimental solubility of NaCl in water, *x*_Cl_,*x*_Na_ ≈ 11%. Indeed, the properties of salts in water are very sensitive to the force field considered (see, e.g., refs (56,57)).

Figure 13c,d shows a snapshot of a WCB containing *N*_0_ = 100 pairs of Na+ and Cl– and at *T* = 320 K, in the presence of carbon dioxide ( = 1502). As discussed above, the NaCl also forms a crystallite within the WCB, avoiding the water–carbon dioxide interface and the wall interface. While the ions are not able to diffuse into the carbon-dioxide volume, some CO_2_ molecules diffuse into the WCB.

**Figure 14:**
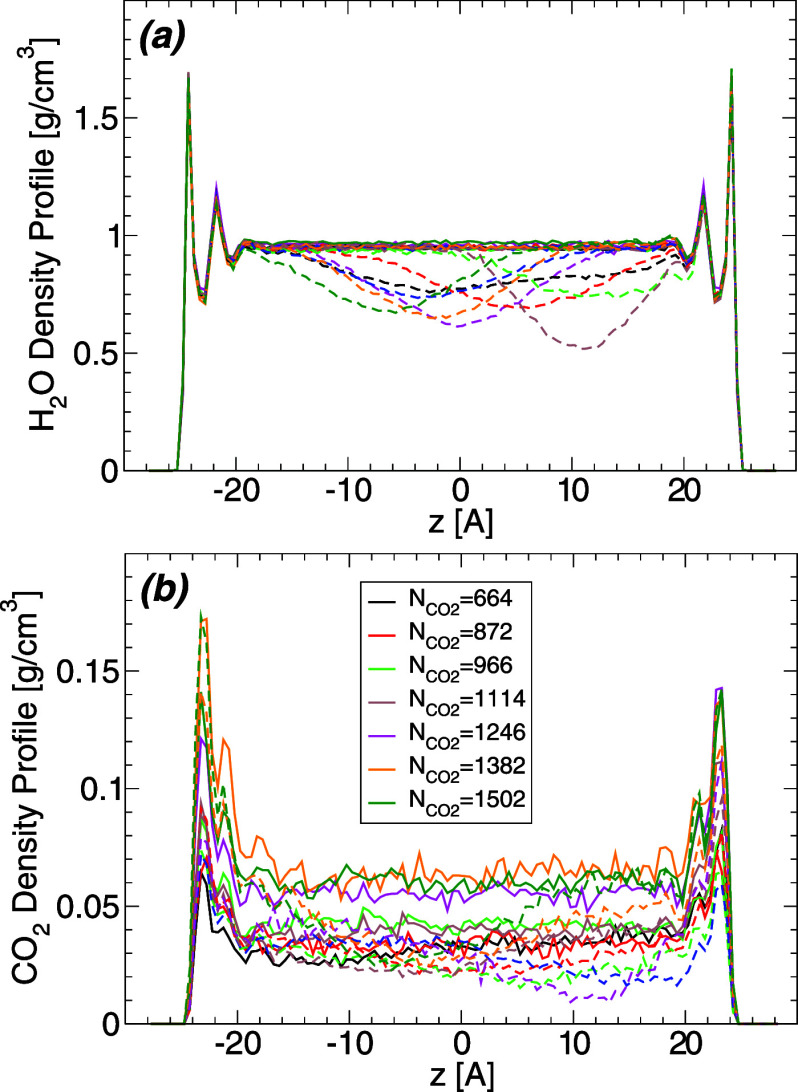
Density profiles of water (a) and carbon dioxide (b) within the WCB containing *N*_0_ = 100 pairs of Na+ and Cl– ions, and along the direction perpendicular to the walls (dashed lines). Results are for *T* = 320 K and different number of CO_2_ molecules. For comparison, we also include the density profiles when the Na+ and Cl– ions are removed from Figure 4a (solid lines).

To confirm the qualitative picture resulting from Figure 13, we also calculate  and . As shown in Figure 14a, the water density profiles are practically independent of the presence of NaCl in the WCB, particularly, at the interfaces with the walls. Interestingly,  develops a pronounced minimum at −20 < *z* < 20 Å when the ions are present. This minimum is due to the NaCl crystallite formed within the WCB. The NaCl crystallite oscillates over time but remains away from the water–wall interface. It is for this reason that  is indifferent to the presence of NaCl close to the walls (|*z*| > 20 Å). Similar conclusions apply to the . The  is weakly affected by the addition of salt; see Figure 14b. In all cases,  is small (<10–20% mass fraction for −20 < *z* < 20 Å). As for the case of water, a weak minimum develops in  at −20 < *z* < 20 Å due to the excluded volume occupied by the NaCl crystallite.

**Figure 15:**
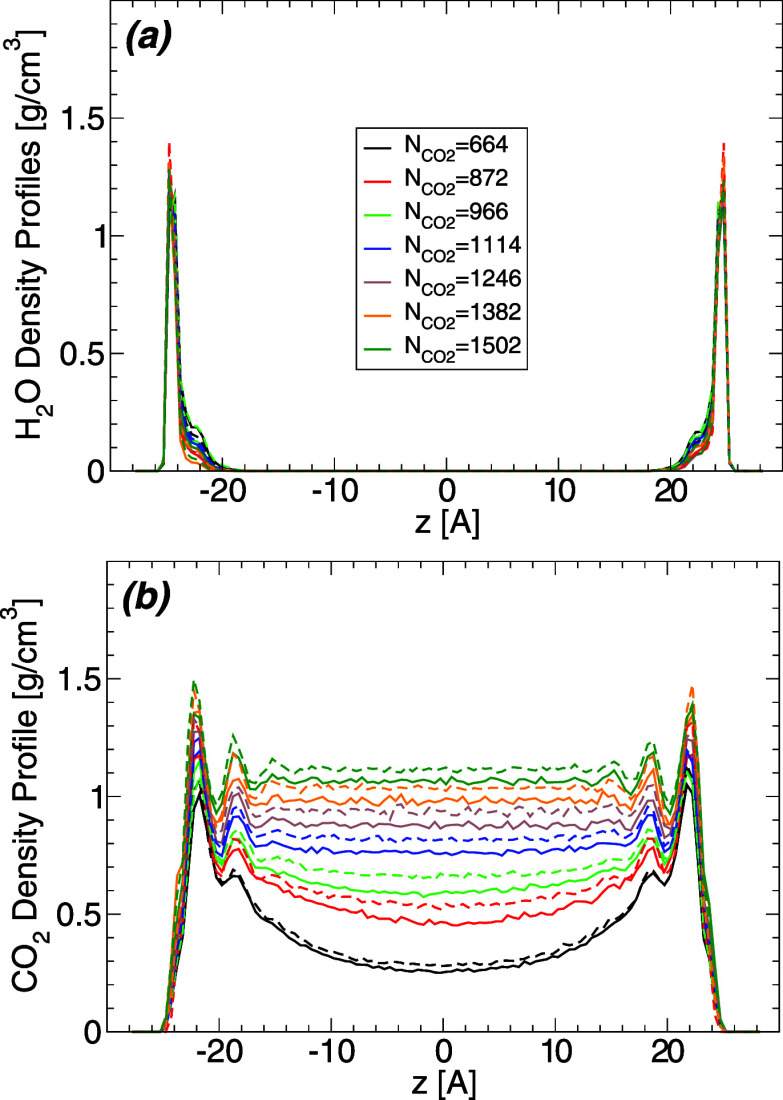
Density profiles of water (a) and carbon dioxide (b) within the CDCB for the case where there are *N*_0_ = 100 pairs of Na+ and Cl– ions in the system (dashed lines). Results are for *T* = 320 K and different numbed of CO_2_ molecules. For comparison, we also include the density profiles when the Na+ and Cl– ions are removed from Figure 4b (solid lines). Both  and  are weakly affected by the presence of NaCl.

One may wonder if the presence of NaCl can affect the distribution of H_2_O and CO_2_ molecules within the carbon dioxide volume. To address this question, we include in Figure 15 the density profiles of H_2_O and CO_2_ along the CDCB. A comparison of the corresponding density profiles with (dashed lines) and without NaCl (solid lines) shows that the  and  exhibit minor changes with the addition of NaCl. It follows that, even in the presence of NaCl, a thin water film is adsorbed on the walls surface. Again, this is because the NaCl ions remain within the WCB and away from the walls and the CDCB.

Interestingly, as shown in Figure 15b, the values of  increase slightly when NaCl is added to the WCB. For example, for the case  = 1502,  increases by 0.04–0.05 g/cm^3^ (≈3–4%) after adding *N*_0_ = 100 pairs of Na+ and Cl– ions. This indicates that the presence of ions decreases slightly the solubility of CO_2_ molecules into the WCB. We note that, as found previously in the absence of NaCl, the surface under the CDCB remains preferentially hydrated by water (as opposed to CO_2_). Qualitative similar results hold when *N*_0_ = 300 pairs of Na+ and Cl– are added to the WCB.

#### Water-and-Salt Capillary Bridges and Contact Angle

**Figure 16:**
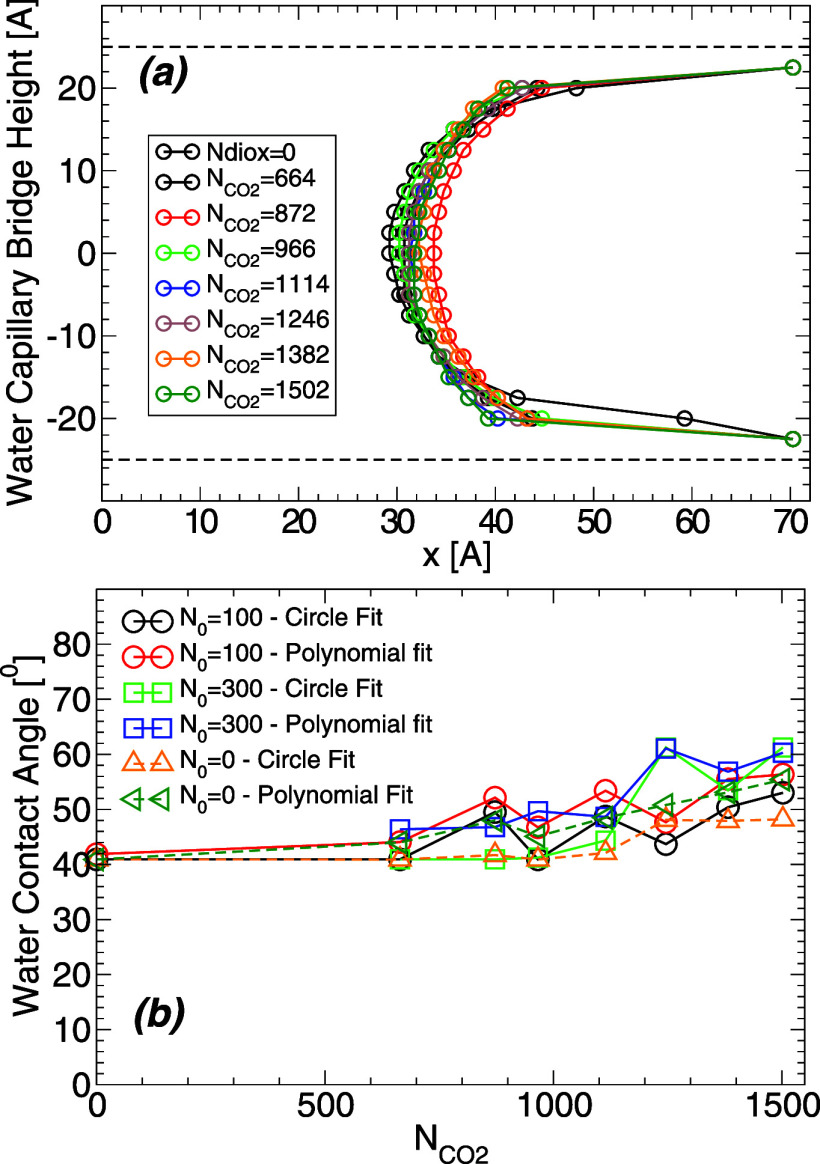
(a) Profile of the WCB containing *N*_0_ = 100 pairs of Na+ and Cl– ions, and surrounded by carbon dioxide; *T* = 320 K (similar results are obtained for *N*_0_ = 300). The dashed lines indicate the location of the walls surface (at *z* = ±25 Å). The density profiles at heights |*z*| = 20–22.5 Å are off due to the presence of water films adsorbed on the walls; these data points do not represent the WCB profile. The effect of increasing the amount of carbon dioxide () on the WCB profile is mild. (b) Water contact angles θ_c_ obtained from (a) by fitting the WCB using a circle and a second order polynomial (black and red lines, respectively). For comparison, also included are the values of θ_c_ in the absence of ions (from Figure 7, orange and dark-green lines) and for the case *N*_0_ = 300 (green and blue lines). θ_c_ increases slightly with increasing amounts of carbon dioxide (Δθ_c_ = 10–20° for  = 0–1502). Within the fluctuations in the data, there is no effect on θ_*c*_ due to the addition of NaCl to the WCB. Estimated error bars are approximately 2–4° (smaller than the symbols size).

In order to explore the effects of adding salt to the wetting behavior of the WCB, we focus on the case *T* = 320 K (supercritical CO_2_) and *N*_0_ = 100 pairs of Cl– and Na+ ions (similar results are obtained for *N*_0_ = 300). Figure 16a shows the average WCB profile (with NaCl) in the presence of  = 0, 664, 872, 966, 1114, 1246, 1382, and 1502 molecules. The effect of increasing the amount of carbon dioxide on the WCB profile is rather mild. The corresponding water contact angles are shown in Figure 16b. The values of θ_c_ fluctuate considerably. Nonetheless, it is apparent that adding NaCl to the WCB does not affect θ_c_. We find an increase of Δθ_c_ = 1–20° with carbon dioxide in the range  = 1502 (supercritical liquid-like carbon dioxide). The changes in θ_c_ are consistent with the observations above that Na+ and Cl– ions are located within the WCB and away from the water–carbon dioxide and water–wall interfaces. We also note that our results, within the fluctuations in the data, seem to be rather independent of whether one uses a circle or a second order polynomial to fit the (salty) WCB profiles.

## Conclusions

In this work, we study the behavior of nanoscale water capillary bridges surrounded by carbon dioxide over a wide range of temperatures and pressures. The water and carbon dioxide system is confined by hydroxylated silica surfaces (β-cristobalite) which can form HB with both H_2_O and CO_2_ molecules. Our simulations show that, consistent with studies based on α-quartz,^40^ our silica walls are preferentially hydrated by water. Accordingly, the carbon dioxide fluid phase in the system is separated from the walls by a thin film of water (1–2 water layers thick). This conclusion holds at all temperatures (*T* = 280–400 K) and pressures studied ( = 0–80 MPa). While the water film adsorbed on the walls is practically insensitive to variations in the CO_2_ content of the system, the water film becomes thicker with increasing temperature. This implies that increasing the temperature favors the release of CO_2_ away from the confining walls. In order to understand why the walls are preferentially hydrated by water, we also perform a molecular-level characterization of the walls hydration. It is found that, relative to the CO_2_ molecules, H_2_O molecules have an enhanced ability to form HB with the surface silanol groups. Specifically, a given water molecule next to the walls is able to form up to three HB with the silanol groups while, instead, most CO_2_ molecules form zero or one HB with the surface. Our MD simulations also show that the WCB contact angle θ_c_ varies weakly with temperature and pressure. For example, Δθ_c_ ≈ + 10–20° for  increasing from ≈0 to 80 MPa (*T* = 320 K), and Δθ_c_ ≈ −10° for *T* increasing from 320 to 360 K (with a fixed amount of carbon dioxide).

The effect of adding salt (NaCl) to the water–carbon dioxide system was also explored at *T* = 320 K (supercritical CO_2_). The MD simulations show that at the salt concentrations studied (mole fractions *x*_Na_ = *x*_Cl_ = 3.50, 9.81%), the NaCl forms a large crystallite within the WCB with the ions avoiding the water–carbon dioxide interface and the walls surface. This results in θ_c_ being insensitive to the presence of NaCl, for all the concentrations of CO_2_ studied ( = 0–80 MPa). Our results on the WCB containing salt are based on the OPLS model for NaCl, and the SPC/E model for water. At the (realistic) concentrations studied, these models predict the aggregation of the ions within the WCB. It would be important in the future to systematically compare the effects of using different models for NaCl, as well as water, on the WCB contact angle. This is important because the solubility of NaCl in water is sensitive to the water–NaCl model considered.^56^

Our results are important for CO_2_ capture and storage technologies. Our MD simulations suggest that the contact angle of water on a hydroxylated silica-based surface, surrounded by carbon dioxide, remains <90° over a wide range of temperature, CO_2_ pressure, and irrespective of salt presence. Hence, caprocks comprised of hydrophilic materials, such as β-cristobalite, should remain water wet, entailing a positive capillary pressure. Using γ = 30 mN/m, θ_c_ = 60° and Φ_P_ = 5 nm in eq 1, we estimate that at least 12 MPa is needed in order for CO_2_ to permeate across the entire caprock layer. Real rocks are obviously mineralogically more complex and heterogeneous than the system modeled in this study so the suitability of caprocks for geological storage should be individually assessed.

## Supporting Information Available

(i) A comparison of the carbon-dioxide equation-of-state predicted by the (flexible) EPM2 model and experiments; (ii) a comparison of the carbon dioxide–water surface tension predicted by the SPC/E water and (flexible) EPM2 carbon dioxide models with experiments; (iii) effects of the surface hydrophobicity/hydrophilicitiy on the (a) carbon dioxide capillary bridges, and (b) water capillary bridges in contact with carbon dioxide; (iv) effects of temperature on the walls hydration; (v) size effects on the water capillary bridge profiles; (vi) additional MD simulations of *bulk* NaCl–water solutions at different concentrations (PDF)
