# Supplementary material for: Water and Carbon Dioxide Capillary Bridges in Nanoscale Slit Pores: Effects of Temperature, Pressure, and Salt Concentration on the Water Contact Angle
Source: Langmuir. 2024 Aug 19;40(35):18439–50. doi: 10.1021/acs.langmuir.4c01185 (PMC11375785; doi:10.1021/acs.langmuir.4c01185)
Supplement: Supplementary file 1 — la4c01185_si_001.pdf [file la4c01185_si_001.pdf]

Supporting Information for "Water and Carbon Dioxide Capillary Bridges in Nanoscale Slit Pores: Effects of Temperature, Pressure, and Salt Concentration on the Water Contact Angle"

Arthur Prado Camargo[†], Arben Jusufi[‡], Alex Gk Lee[*,‡], Joel Koplik[¶,§,‖], Jeffrey F. Morris[*,¶,⊥,‖], and Nicolas Giovambattista[*,#,‖]

† *Instituto de Física, Universidade de São Paulo, Rua do Matão, 1371 CEP 05508-090, Brasil*

‡ *ExxonMobil Technology and Engineering Company, 1545 US Rt. 22 East, Annandale, NJ 08801, United States*

¶ *Levich Institute, City College of New York, New York, NY 10031, United States*

§ *Department of Physics, City College of New York, New York, NY 10031, United States*

‖ *Ph.D. Program in Physics, The Graduate Center of the City University of New York, New York, NY 10016, United States*

⊥ *Department of Chemical Engineering, City College of New York, New York, New York 10031, United States*

\# *Department of Physics, Brooklyn College of the City University of New York, Brooklyn, New York 11210, United States*

**E-mail: alex.gk.lee@exxonmobil.com; morris@ccny.cuny.edu; ngiovambattista@brooklyn.cuny.edu*

S1) Validation of the Carbon Dioxide Force Field: 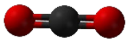

We compare the experimental pressure-density equation-of-state of (bulk) carbon dioxide with the corresponding results obtained from molecular dynamics (MD) simulations using three CO_2 force fields, the TraPPE model[1], EPM2[2], and *flexible* EPM2 model[3]. The TraPPE model is rigid, i.e., it assumes that the CO covalent bond length and OCO angle are fixed, while the "flexible EPM2" model does not. In the "EPM2 model"[2], only the CO covalent bond length is fixed.

Fig. S1 shows the P(ρ)-equation of state obtained from our MD simulations of a cubic box of CO_2 molecules using the three force fields mentioned above. All force fields considered are able to reproduce well the P(ρ)-equation of state from experiments.

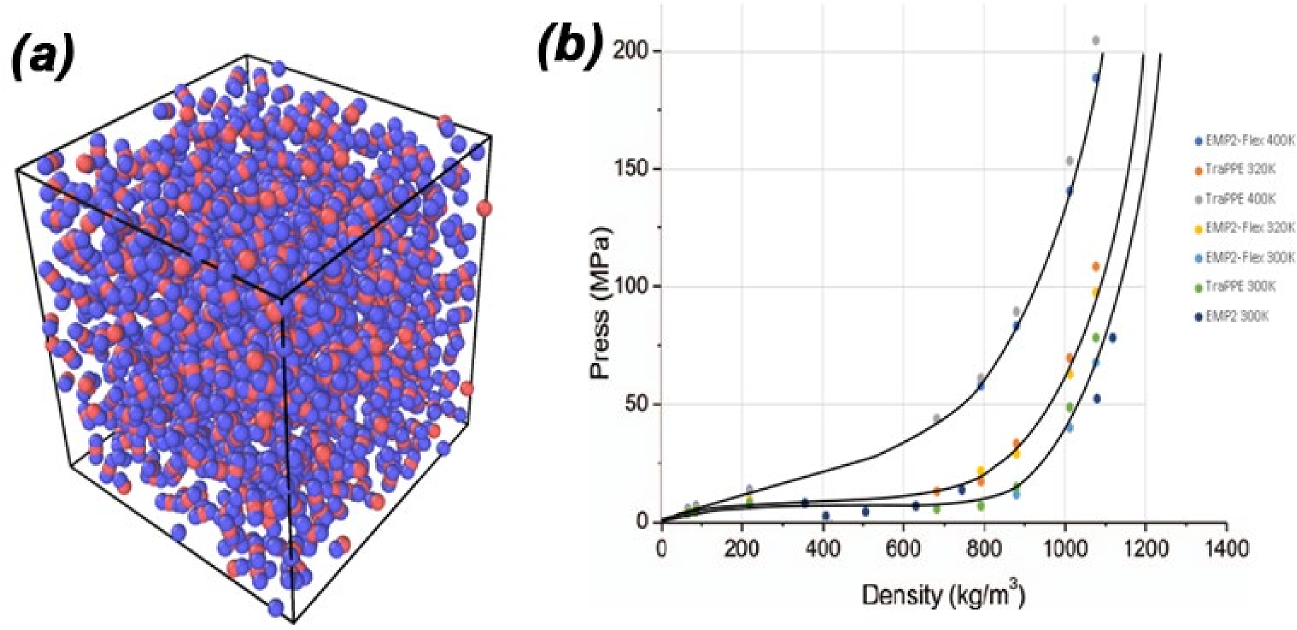

Figure S1. (a) Snapshot of a cubic box containing carbon dioxide molecules (the system is periodic along the three directions); blue and red spheres represent the O and C atoms. (b) P(ρ)-equation of state obtained from our MD simulations of carbon dioxide using different force fields (symbols). Lines correspond to experimental data from NIST obtained at approximately T=400, 320, and 300 K (top-to-bottom).

S2) Validation of the interfacial tension of SPC/E water and (flexible) EPM2 carbon dioxide

For interfacial tension calculations between SPC/E water and (flexible) EPM2 carbon dioxide, we generated initial configurations of a corresponding two-phase system in an orthogonal box, one phase containing pure water and the other containing pure CO_2. The simulation box dimensions are (5.2×5.2×14.0) nm^3 with imposed periodic boundary conditions; the two phases are separated in the z-direction. The number of water molecules was set to N=2756 whereas the number of CO_2 molecules was varied to control the pressure. A series of simulation stages were performed for each system. After a short energy minimization, MD runs were performed at a temperature of 323 K (50 °C) for 0.25 ns with a simulation timestep of 0.5 fs. The temperature was kept constant using a Nosé-Hoover thermostat with a relaxation time of 100 timesteps[5,6]. A subsequent MD run was carried out, this time for 20 ns with a simulation timestep of 1 fs; the other parameters were kept unchanged. During the last 10 ns, we calculated the interfacial tension and the average density of CO_2 at T=50 °C. Due to the solubility of CO_2 in water, the average density is lower than the initial CO_2 density. We use the average density to calculate the CO_2 pressure using the equation of state for CO_2, described in Section S1 and Fig. 6 of the main manuscript. The interfacial tension, γ, was calculated from the principal stress tensor components, $-P_{ii}$ (where *i=x, y, z*), to obtain the difference between the lateral and perpendicular pressure components acting on the cross-sectional interface spanning the x-y plane:

$$\gamma = L_z \langle P_{zz} - \frac{1}{2}\left(P_{xx} + P_{yy}\right)\rangle,$$

where L_z = 14 nm being the box size in z, and $\langle \ldots \rangle$ denoting time averages.

The dependency of the interfacial tension on the CO_2 pressure is shown in Figure S2. Plotted are two sets of simulation results, one in the absence of salt (red line) and one in the presence of 1.5 M NaCl (blue line). Although the experimental data in the absence of salt (*m=0.0*) are noisy, the MD values of γ are consistent with the experiments at intermediate and high pressures. At low pressures, our MD simulations underestimate the experimental values of γ which is not surprising in view of the deficiency of the SPC/E water model in capturing the surface tension of water[7]. The entire behavior shown in Figure S2 agrees well with recently reported MD results in which various water/CO_2 models were tested, including the ones used in this work[8].

In the presence of salt (*m>0.0*), the MD simulations underestimate the experimental values of γ at all pressures studied. In addition, our MD simulations show that γ is independent of the NaCl concentration. While this is consistent with experiments at low pressure, at P<5 MPa, the experimental data in Fig. S2 at high pressure (at P>5 MPa) shows that, at a given pressure, the interfacial tension γ increases with increasing salt concentration, *m* (see the data by Li et al.[16,17] in Fig. S2). The inability of our MD simulations to capture the dependence of γ with the salt concentration may be due to the water model and/or NaCl force field employed. In our MD simulations, we observe the formation of NaCl crystallites within the water film, like the crystallites found in the WCB (Fig. 13 of the main manuscript). These crystallites and hence, the NaCl ions, remain away from the liquid-vapor interface which is consistent with γ being independent of the salt concentration (in the MD simulations). We note that NaCl cluster formation had been reported before for different NaCl forcefields and water models[9,10].

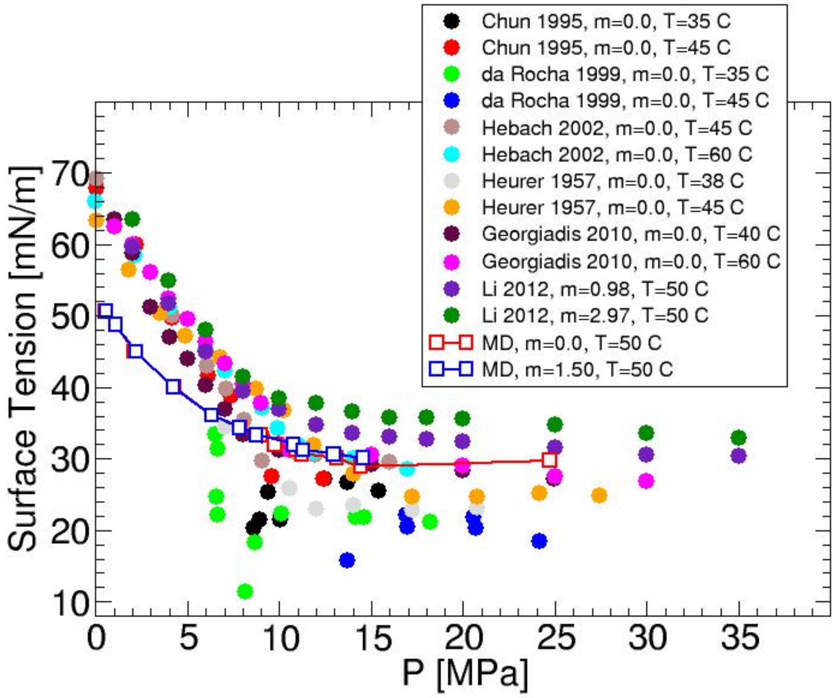

Figure S2. Interfacial tension between water and CO_2 as a function of the CO_2 pressure. MD simulation results were calculated at 50 °C in the presence (*m=1.50*) and absence (*m=0.0*) of NaCl (empty blue and red squares) and are compared to experimental data obtained from various sources, as noted in the legends (solid circles). Experimental data is from Refs.[11,12,13,14,15,16,17].

S3) Carbon dioxide capillary bridges formed between hydrophobic "β-cristobalite" walls

The β-cristobalite surface that we use in the MD simulations is hydrophilic; see Figs. 2a and 3a in the main manuscript. This is due to the formation of hydrogen bonds (HB) between the walls silanol groups and the water molecules. Accordingly, when the partial charges of the walls silanol groups are removed (and hence, the H atoms of the walls are effectively removed[4]) the walls become hydrophobic; see Fig. 3b in the main manuscript.

In the case of carbon dioxide, the original β-cristobalite surfaces are solvophilic, i.e., appealing to CO_2 molecules; see Fig. 3(c)(d). However, we find that when the partial charges of the wall silanol groups are removed (and hence, the H atoms of the walls are effectively removed), the walls remain solvophilic to carbon dioxide; see Fig. S3. This implies that, contrary to the case of water, the walls are appealing to CO_2 not only due to the formation of walls-CO_2 hydrogen-bonds but also due to the wall-CO_2 Lennard-Jones (LJ) interactions. This can be understood by noticing that there is only one LJ pair interaction between a given water molecule and a Si/O atom in the walls (i.e, between the water O atom and the wall Si/O atom) while there are three LJ pair interactions between a given CO_2 molecule and a Si/O atom in the walls (i.e., between the carbon dioxide molecule C and two O atoms, and the wall Si/O atom).

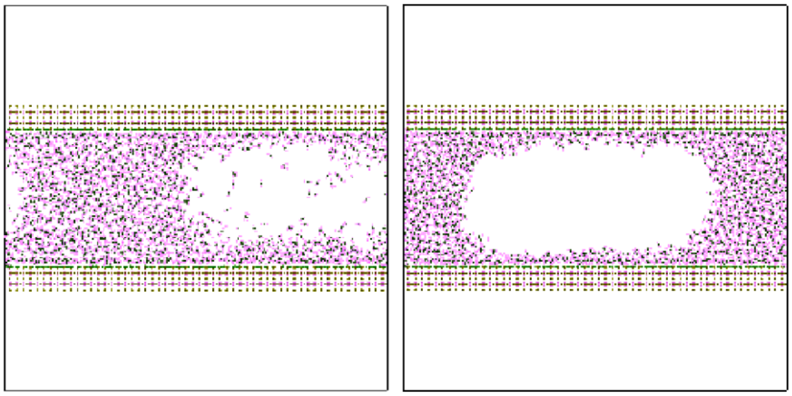

Figure S3. Carbon dioxide capillary bridges formed between β-cristobalite surfaces separated by h=5 nm; no water molecule is present (N_{CO2}=1670). All partial charges at the surfaces' silanol groups are removed (effectively removing the walls H atoms as well). While these walls are hydrophobic (i.e., water repellent), the walls remain solvophilic to CO_2. At T=240 K (left panel) and 150 K (right panel) liquid carbon dioxide forms films covering the whole surface of the walls. The CO_2 capillary bridge forms on top of the adsorbed films.

S4) Water capillary bridges and carbon dioxide nanoconfined by *hydrophobic* "β-cristobalite"

We compare the water capillary bridges (WCB) formed between the original β-cristobalite surfaces (top row in Fig. S4) with the corresponding WCB formed between the same β-cristobalite surfaces after the partial charges of the walls are removed and the silanol H atoms effectively disappear (bottom row in Fig. S4). As shown in Fig. S4, the β-cristobalite surfaces become hydrophobic once the wall partial charges are removed (i.e., the surface of the WCB becomes convex and the contact angle of water is >90^0). However, the walls remain solvophilic to CO_2 (see

Sec. S3 above). Consistent with these observations, the snapshots in the bottom row of Fig. S4 show a film of carbon dioxide covering the whole surface of the walls. In particular, the film of carbon dioxide extends under the WCB.

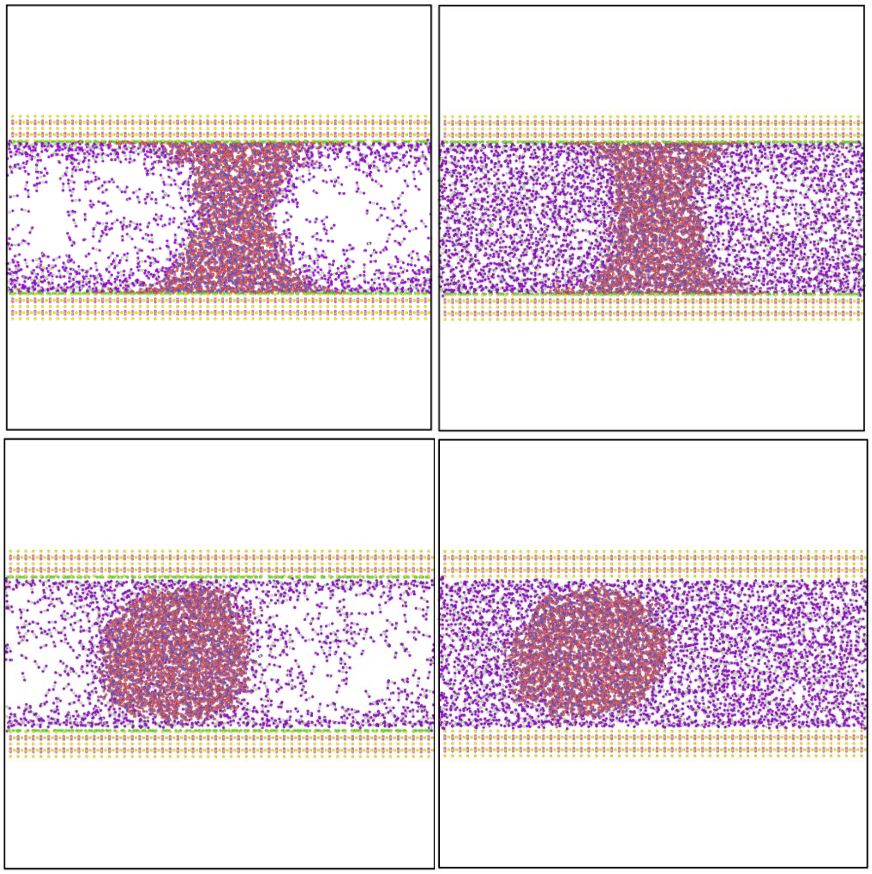

Figure S4. Top row: WCB formed between the original β-cristobalite surfaces. The WCB has N=1144 water molecules and there are N_{CO2}=800, 1670 carbon dioxide molecules (left-to-right). Bottom row: same as top row but for the case of β-cristobalite surfaces with all partial charges being removed. In this case, the β-cristobalite surfaces become hydrophobic (water repellent) but remain solvophilic (appealing) to carbon dioxide. Note the film of carbon dioxide covering the walls and separating the WCB from the walls surface (bottom row).

S5) Effects of temperature on the walls hydration for the case N_{CO2}=1502

Included in Fig. S5 are additional density profiles for H_2O and CO_2 within the WCB, $\rho_{H2O,W}(z)$ and $\rho_{CO2,W}(z)$, at different temperatures. Results are for the case N_{CO2}=1502 carbon dioxide molecules (N=2756 water molecules). The corresponding density profiles for H_2O and CO_2 within the CDCB, $\rho_{H2O,CD}(z)$ and $\rho_{CO2,CD}(z)$, are included in Fig. S6. Figs. S5 and S6 are analogous to Figs. 5a and 5b for the case N_{CO2}=1114 (N=2756).

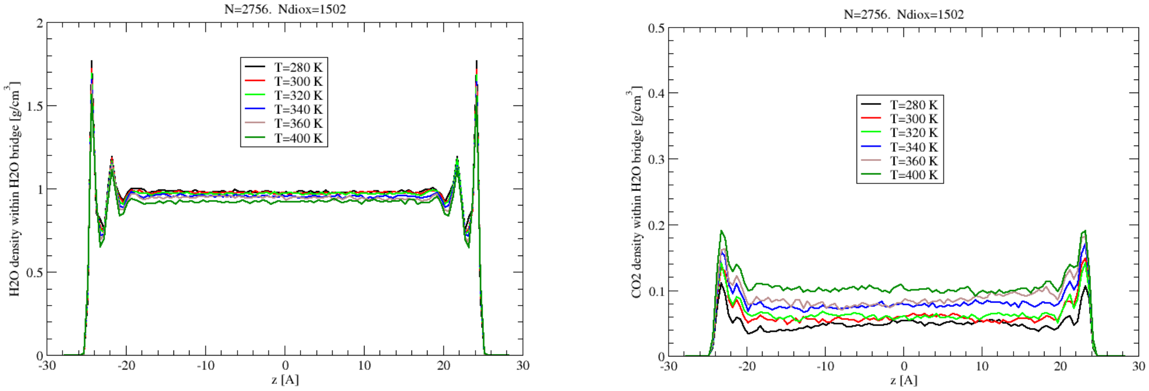

Figure S5. Same as Fig. 5a of the main manuscript for the case N_{CO2}=1502 (N=2756). Density profiles of water (left) and carbon dioxide (right) within the WCB, and along the direction perpendicular to the walls. Varying the temperature changes slightly the local density of water and carbon dioxide within the WCB but it does not affect the location of the water/carbon dioxide layers close to the walls.

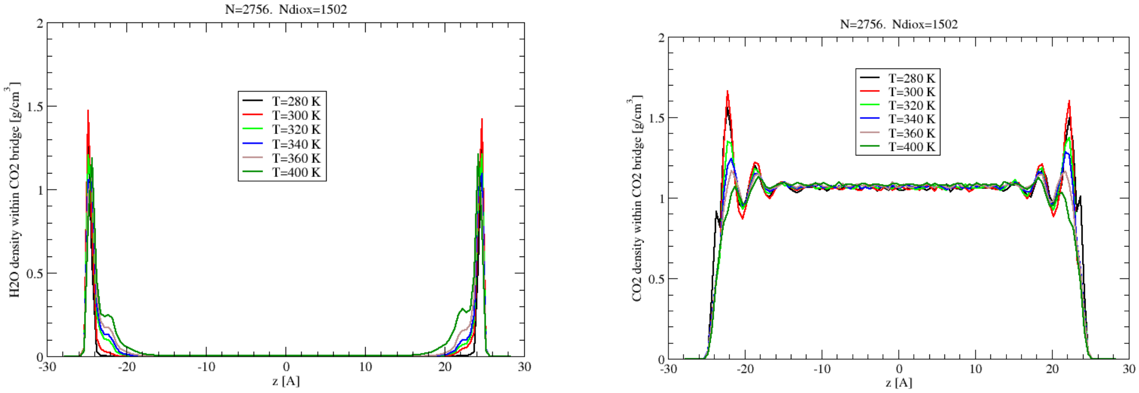

Figure S6. Same as Fig. 5b of the main manuscript for the case N_{CO2}=1502 (N=2756). Density profiles of water (left) and carbon dioxide (right) within the carbon-dioxide capillary bridge (CDCB), and along the direction perpendicular to the walls. Varying the temperature changes slightly the local density of water and carbon dioxide within the CDCB increasing the number of water molecules in the water films adsorbed at the walls surfaces and pushing the CO_2 molecules away from the walls (towards the central region (z=0) of the CDCB).

S6) Size effects on the water capillary bridge profiles

The results in the main manuscript are based on WCB containing N=2756 water molecules. In Fig. S7 we show results obtained with WCB composed of N=1140 water molecules. Fig. S7 strongly indicates that there are negligible size-effects, if any, in our results.

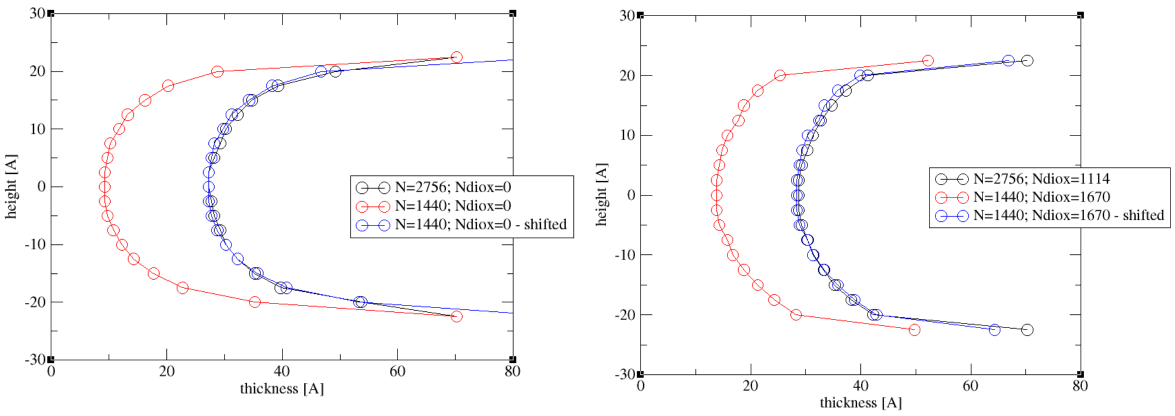

Figure S7. WCB profiles from MD simulations where the WCB contain N=2756 (black line) and N=1140 (red line) water molecules; T=320 K. Left: WCB with no carbon dioxide; right: WCB surrounded by N_{CO2}=1670 CO_2 molecules. The blue line is the WCB profile obtained by shifting the WCB profile for N=1140 (red line) along the x-axis. The overlap between the blue and black lines indicate that size-effects are negligible, if any.

S7) Crystallization of NaCl in bulk water

We also performed MD simulations of *bulk* water (N=3375) with salt (NaCl) at concentrations x_{Cl}=x_{Na}= 0.91, 1.80, 4.93, 9.40 % to study the crystallization of NaCl at T=300 K and P=0.1 MPa (N_0=31, 62, 175, and 350 NaCl pairs, respectively). As shown in Fig. S8, crystallites are found at mole fractions x_{Cl}=x_{Na}=4.93, 9.40 % corresponding to the addition of N_o=143, 286 NaCl ion pairs in our WCB (N=2756); crystallites are not observed at x_{Cl}=x_{Na}= 0.91, 1.80 % corresponding to the addition of N_0=25, 50 NaCl pairs in our WCB (N=2756).

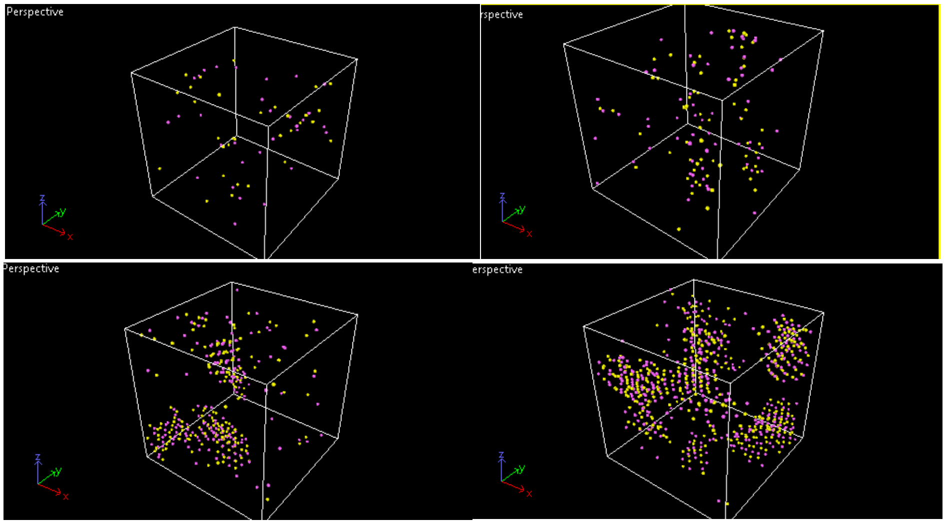

Figure S8. Snapshots from MD simulation of water (N=3375) containing N_0=31, 62, 175, and 350 pairs of Cl^- and Na^+ [only the Na and Cl ions are shown].

****

REFERENCES:

[1] Trinh, T.; Vlugt, T.; Kjelstrup, S. Thermal conductivity of carbon dioxide from non-equilibrium molecular dynamics: A systematic study of several common force fields. *J. Chem. Phys.* **2014**, *141*, 134504.

[2] Harris, J.; Yung, K. Carbon Dioxide's Liquid-Vapor Coexistence Curve And Critical Properties as Predicted by a Simple Molecular Model. *J. Phys. Chem*. **1995**, *99*, 12021.

[3] Sun, E.; Bourg, I. Molecular Dynamics Simulations of Mineral Surface Wettability by Water Versus CO2: Thin Films, Contact Angles, and Capillary Pressure in a Silica Nanopore*. J Phys. Chem. C* **2020**, *124*, 25382.

[4] Giovambattista, N.; Rossky, P. J.; Debenedetti, P. G. Effect of pressure on the phase behavior and structure of water confined between nanoscale hydrophobic and hydrophilic plates. *Phys. Rev. E* **2006**, *73*, 041604.

[5] Nosé, S. A molecular dynamics method for simulations in the canonical ensemble. *Mol. Phys.* **1984**, *52*, 255-268.

[6] Hoover, W. G. Canonical dynamics: Equilibrium phase-space distributions. *Phys. Rev. A* **1985**, *31*, 1695-1697.

[7] Vega, C.; de Miguel, E. Surface tension of the most popular models of water by using the test-area simulation method. *J. Chem. Phys.* **2007**, *126*, 154707.

[8] Shiga, M.; Morishita, T.; Sorai, M. Interfacial tension of carbon dioxide - water under conditions of CO_2 geological storage and enhanced geothermal systems: A molecular dynamics study on the effect of temperature. *Fuel* **2023**, *337*, 127219.

[9] Patra, M.; Karttunen, M. Systematic comparison of force fields for microscopic simulations of NaCl in aqueous solutions: diffusion, free energy of hydration, and structural properties. *J. Comput. Chem.* **2004**, *26*, 678-689.

[10] Lenart, P. J.; Jusufi, A.; Panagiotopoulos, A. Z. Effective potentials for 1:1 electrolyte solutions incorporating dielectric saturation and repulsive hydration. *J. Chem. Phys.* **2007**, *126*, 044509.

[11] Chun, B.; Wilkinson, G. T. Interfacial Tension in High-Pressure Carbon Dioxide Mixture. *Ind. Eng. Chem. Res.* **1995**, *34*, 4371-4377.

[12] da Rocha, S.; Harrison, K. L.; Johnston, K. P. Effect of Surfactants on the Interfacial Tension and Emulsion Formation between Water and Carbon Dioxide. *Langmuir* **1999**, *15*, 419-428.

[13] Hebach, A.; Oberhof, A.; Dahmen, N.; Kogel, A.; Ederer, H.; Dinjus, E. Interfacial Tension at Elevated Pressure Measurements and Correlations in the Water + Carbon Dioxide System, *J. Chem. Eng. Data* **2002**, *47*, 1540-1546.

[14] Heuer, G. J. Interfacial Tension of Water Against Hydrocarbon and Other Gases and Adsorption of Methane and Solids at Reservoir Conditions, PhD Thesis, The University of Texas at Austin (1957).

[15] Georgiadis, A.; Maitland, G.; Trusler, J. P. M.; Bismarck, A. Interfacial tension measurements

of the (H_2O+ CO_2) system at elevated pressures and temperatures. *J. Chem. Eng. Data* **2010***,* *55*, 4168-4175.

[16] Li, X.; Boek, E.; Maitland, G. C.; Trusler, J. M. Interfacial tension of (Brines+ CO2):(0.864 NaCl+ 0.136 KCl) at temperatures between (298 and 448) K, pressures between (2 and 50) MPa, and total molalities of (1 to 5) mol·kg^{-1}, *J. Chem. Eng. Data,* **2012***, 57*, 1078-1088.

[17] Li, X.; Boek, E.; Maitland, G. C.; Trusler, J. M. Interfacial tension of (Brines+ CO2): CaCl2 (aq), MgCl2 (aq), and Na2SO4 (aq) at temperatures between (343 and 423) K, pressures between (2 and 50) MPa, and molalities of (0.5 to 5) mol·kg^{-1}, *J. Chem. Eng. Data* **2012***, 57*, 1369-1375.
